# Genetic Analysis of Baker's Yeast Msh4-Msh5 Reveals a Threshold Crossover Level for Meiotic Viability

**DOI:** 10.1371/journal.pgen.1001083

**Published:** 2010-08-26

**Authors:** K. T. Nishant, Cheng Chen, Miki Shinohara, Akira Shinohara, Eric Alani

**Affiliations:** 1 Department of Molecular Biology and Genetics, Cornell University, Ithaca, New York, United States of America; 2 Institute for Protein Research, Osaka University, Suita, Osaka, Japan; The University of North Carolina at Chapel Hill, United States of America

## Abstract

During meiosis, the Msh4-Msh5 complex is thought to stabilize single-end invasion intermediates that form during early stages of recombination and subsequently bind to Holliday junctions to facilitate crossover formation. To analyze Msh4-Msh5 function, we mutagenized 57 residues in *Saccharomyces cerevisiae* Msh4 and Msh5 that are either conserved across all Msh4/5 family members or are specific to Msh4 and Msh5. The Msh5 subunit appeared more sensitive to mutagenesis. We identified *msh4* and *msh5* threshold (*msh4/5-t*) mutants that showed wild-type spore viability and crossover interference but displayed, compared to wild-type, up to a two-fold decrease in crossing over on large and medium sized chromosomes (XV, VII, VIII). Crossing over on a small chromosome, however, approached wild-type levels. The *msh4/5-t* mutants also displayed synaptonemal complex assembly defects. A triple mutant containing a *msh4/5-t* allele and mutations that decreased meiotic double-strand break levels (*spo11-HA*) and crossover interference (*pch2Δ*) showed synergistic defects in spore viability. Together these results indicate that the baker's yeast meiotic cell does not require the ∼90 crossovers maintained by crossover homeostasis to form viable spores. They also show that Pch2-mediated crossover interference is important to maintain meiotic viability when crossovers become limiting.

## Author Summary

In meiosis, sex cells that become eggs or sperm undergo a single round of DNA replication followed by two consecutive chromosomal divisions. In most organisms, the segregation of chromosomes at the first meiotic division is dependent upon at least one genetic exchange, or crossover event, between homologous chromosome pairs. Matched chromosomes that do not receive a crossover frequently undergo non-disjunction at the first meiotic division, yielding gametes that lack chromosomes or contain additional copies. Such missegregation events have been linked to Down syndrome and human infertility. This paper focuses on Msh4-Msh5, a complex required for the proper segregation of homologous chromosomes during the Meiosis I division. We performed a mutational analysis of the baker's yeast Msh4-Msh5 complex to study its role in implementing the decision to make a crossover. We identified a class of mutants that are functional in meiosis despite significant reductions in crossing over that occurred primarily on larger chromosomes. In combination with mutations (*pch2Δ*, *spo11-HA*) that disrupted early steps in crossover placement, this *msh4/5* class of mutants displayed poor spore viability. Together, these data support the presence in yeast of a robust crossover distribution mechanism.

## Introduction

Meiosis produces haploid gametes from diploid progenitor cells. This reduction in ploidy results from the segregation of homologous chromosomes at the first meiotic division (Meiosis I). In most organisms, the accurate segregation of chromosomes during Meiosis I requires crossing over between homologs. These crossovers provide physical linkages between homologs that enable their proper positioning at metaphase I through spindle microtubule generated forces [1]. Disruption of these forces by the loss of chromosome arm cohesion facilitates the Meiosis I division [2]. Failure to achieve at least one crossover per homolog pair results in non-disjunction of the homolog pair, leading to the production of aneuploid gametes (reviewed in [3]).

Meiotic crossing over is initiated in meiotic prophase by the formation of Spo11-dependent DNA double strand breaks (DSBs; [4]). Meiotic DSBs can be repaired as either crossovers or non-crossovers through distinct repair pathways [5], [6]. In *Saccharomyces cerevisiae*, approximately 60% of the 140–170 DSBs that form in meiosis (estimated from a whole genome microarray analysis of *dmc1Δ* and *dmc1Δ rad51Δ* mutants) are processed as crossovers [7], [8]. A single *S. cerevisiae* cell in meiosis forms approximately 90 crossovers distributed over sixteen homolog pairs [9]–[11]. In contrast, in *C. elegans* meiosis, only a single crossover forms between each homolog pair that ensures Meiosis I disjunction [12].

The majority of meiotic crossovers in baker's yeast display interference. Interference ensures that a crossover designation for one DSB site makes a non-crossover fate more likely at adjacent sites, and leads to the formation of widely and evenly spaced crossovers [13]–[15]. In the interference-dependent crossover pathway, DSBs are processed to form single end invasion intermediates (SEIs) that result from the invasion of a DSB end into an intact homolog. These intermediates are then thought to undergo second-end capture with the intact homolog to form double Holliday junctions (dHJs) that are ultimately resolved to form crossovers [16]–[19]. A crossover homeostasis mechanism was identified in baker's yeast that ensures crossovers are preferentially formed at the expense of non-crossovers when the number of initiating DSBs is reduced [20]. Thus crossover interference and homeostasis ensure formation of at least one crossover on all homolog pairs [20], [21]. The presence of at least one crossover per homolog pair is known as the obligate crossover. Barchi *et al.* [22] further define the obligate crossover “as one of the outcomes of the process(es) through which most crossovers form, not as a special type of crossover.” Control mechanisms that ensure the obligate crossover are likely to act during the crossover/non-crossover decision, an event that takes place at or just prior to SEI formation [5], [6]. It is important to note that previous work in baker's yeast suggested that ∼20% of crossovers on a large chromosome and ∼50% of crossovers on a small chromosome involved interference-independent crossovers that occurred through a distinct Mms4-Mus81 pathway [23], [24].

**Figure 1:**
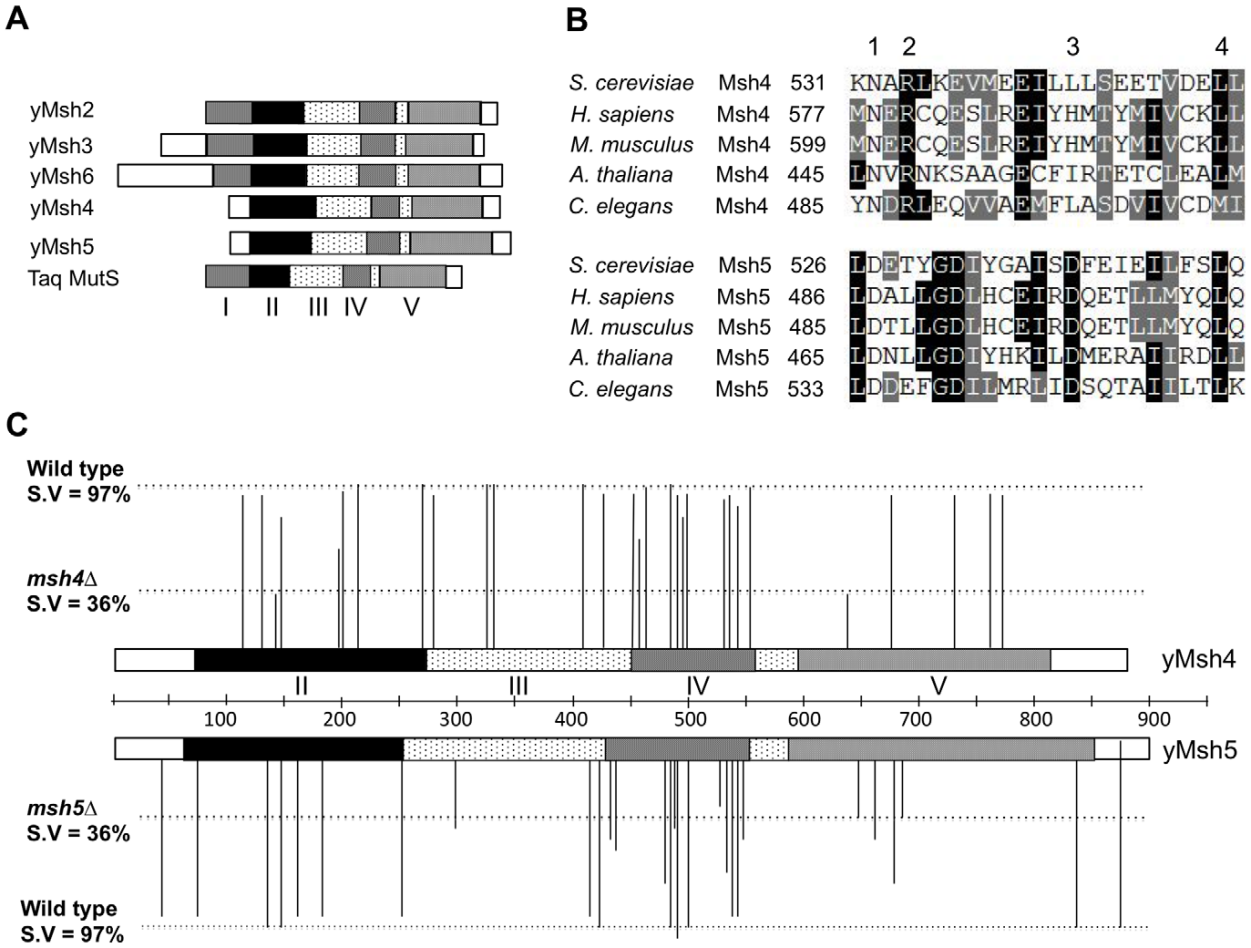
Structure-function analysis of *msh4*, *msh5* alleles. (A) Comparison of domain organization of yeast Msh proteins with the *Thermus aquaticus* (*Taq*) MutS protein. The five domains (I–V) identified in yeast Msh proteins based on structural homology to *Taq* MutS are shown to scale [35]. (B) Sequence alignment of Msh4 protein sequences from *S. cerevisiae* (YFL003C), *H. sapiens* (NM_002440), *M. musculus* (BC145838), *A. thaliana* (NM_117842) and *C. elegans* (AF178755) and Msh5 protein sequences from *S. cerevisiae* (YDL154W), *H. sapiens* (BC002498), *M. musculus* (NM_013600), *A. thaliana* (EF471448) and *C. elegans* (NM_070130). Representative residues from four different classes used for structure-function analysis are shown; Class 1 (Msh4, Msh5 specific); Class 2 (Msh4 specific); Class 3 (Msh5 specific) and Class 4 (Msh family specific). (C) Spore viability profiles of 57 *msh4*, *msh5* mutations in the EAY background are shown with reference to the domain organization of the Msh4, Msh5 proteins. The height of each line corresponds to the spore viability of each mutant relative to wild-type and null. Four domains (II–V) in the Msh4, Msh5 proteins based on structural homology to MutS are shown [35].

The ZMM proteins (Zip1-4, Spo16, Mer3, Msh4-Msh5) act as pro-crossover factors in the interference-dependent crossover pathway by coordinating crossing over with formation of the synaptonemal complex, a zipper-like structure that connects homologous chromosomes in late stages of meiotic prophase [24]–[34]. Msh4-Msh5 attracted our attention because strains defective in this complex show strong defects in Zip1 polymerization during synaptonemal complex formation [27], [29]. Msh4 and Msh5 each contain domains II–V found in the bacterial MutS family of mismatch repair proteins, but lack the N- terminal domain I that is required to interact with domain IV for mismatch DNA binding (Figure 1A; [25], [26], [35], [36]). *S. cerevisiae msh4Δ* and *msh5Δ* mutants display reduced crossing over (∼2.5 fold decreased) and spore viability (30–40%). Tetrads obtained from these mutants display an excess of zero and two viable spores compared to wild-type. This phenotype is consistent with a Meiosis I disjunction defect [24]–[27]. The equivalent mutations in male and female mice result in sterility as a consequence of chromosome pairing and synapsis defects [37]–[39]. The residual crossovers seen in yeast *msh4/5Δ* mutants lack genetic interference [24], [27]; however in *msh4Δ* mutants, Zip2 foci, which mark crossover designation sites, still display a pattern indicating that they are subject to interference [40]. These and other data suggest that Msh4-Msh5 acts after the crossover/noncrossover decision [16], [40]. Consistent with the above data, biochemical and molecular studies showed that Msh4-Msh5 is required to stabilize SEIs and is capable of specifically binding to Holliday junctions as multiple sliding clamps [16], [41].

Additional cell biological observations, primarily in the mouse, have led to a model in which Msh4-Msh5 interacts with the MutL mismatch repair homologs Mlh1-Mlh3 to resolve Holliday junctions [25], [41]–[47]. In mouse spermatocytes in zygotene, Msh4/5 foci are present at high levels (∼140 per nucleus) but decrease until mid pachytene, where they are present at roughly twice the number of crossover sites. At this stage, roughly half of Msh4/5 foci interact with Mlh1/3 foci, which localize to sites of crossing over [48]–[50]. The presence of a large number of Msh4/5 foci in zygotene suggest the possibility of early roles for Msh4/5 in meiosis; consistent with this idea is work in *Sordaria* which show an early role for Msh4-Msh5 during interhomolog interactions, at a time prior to when it is required for recombination progression [51].

The above information encouraged us to systematically mutagenize Msh4-Msh5 to study its role in implementing the crossover decision. We identified a class of *msh4/5* threshold (*msh4/5-t*) mutants that displayed high spore viability despite 1.5 to 2 fold reductions in crossing over that occurred primarily on large (XV, VII) and medium (VIII) sized chromosomes. *msh4/5-t* mutants displayed Msh5 foci similar to wild-type; however, they showed defects in Zip1 polymerization during synaptonemal complex formation. This phenotype is consistent with defects in a crossover maturation process that occurs after Msh4-Msh5 loading onto chromosomes. A triple mutant containing a *msh4/5-t* allele and mutations that decreased DSB levels (*spo11-HA*) and crossover interference (*pch2Δ*) showed preferential loss of crossovers on the small chromosome III and a synthetic spore viability defect, suggesting that crossover interference is critical to maintain meiotic viability when crossovers become limiting.

## Results

### Rationale for structure-function analysis of Msh4 and Msh5

Msh4 and Msh5 amino acid sequences from *S. cerevisiae*, *H. sapiens*, *M. musculus*, *A. thaliana*, and *C. elegans* were aligned using clustalW and CLC free Workbench software (Figure 1, Figure S1; data not shown). We selected four different classes of conserved residues to alter by site-specific mutagenesis (Figure 1B). Class 1 (Msh4/5-specific) residues were conserved in Msh4 and Msh5 but were not conserved in other Msh family members such as Msh2, Msh3, and Msh6. Class 2 (Msh4-specific) and Class 3 (Msh5-specific) were conserved only in Msh4 and Msh5, respectively (Figure 1B; Table 1). Previous work by Pochart *et al.* [52] showed that mutations in the ATP binding domain of Msh5 conferred a null phenotype. Based on these observations, we also mutagenized ATP and DNA binding residues conserved among all Msh family members (Class 4). Eight of these Class 4 mutations were in homologous positions in Msh4 and Msh5 (Figure S1). In total 57 residues were mutated, 29 from Msh4 and 28 from Msh5 (Table 1). All residues were mutated to alanine, with the exception of one residue in the Msh4/5 ATP binding domain that was mutated to tryptophan to allow comparison with an amino acid substitution in a homologous position in Msh2 that affected Msh2-Msh6 ATP hydrolysis [53]. All alleles were integrated into the congenic SK1 strain EAY1108 (EAY background, [24]).

**Table 1:** Spore viability and genetic map distances in EAY1108/EAY1112 strains bearing the indicated *msh4* and *msh5* mutations.

Allele	(Class, Domain)	n	S.V. (%)	Total Rf (cM)	Yeast two hybrid β galactosidase units
*Wild-type*		199	97.0	96.1	54±3.7
*msh4Δ*		557	35.9	39.2	
*msh5Δ*		3990	36	37	
*msh4-E111A*	(2, II)	117	93.4	80.5	
*msh4-N126A*	(1, II)	109	91.7	81.1	
*msh4-D139A*	(2, II)	100	31	38.6	1.5±0.36
*msh4-Y143A*	(2, II)	118	76.1	41.5	
*msh4-F194A*	(1, II)	120	56.7	44.1	
*msh4-N195A*	(2, II)	120	95.4	87.6	
*msh4-D210A*	(1, II)	120	96.9	74.2	
*msh4-D268A*	(1, II)	120	95	70.7	
*msh4-E276A*	(2, II)	180	88.9	53.2	70±30
*msh4-E324A*	(2, III)	119	95.2	101.6	
*msh4-E328A*	(2, III)	119	95.4	83.9	
*msh4-N409A*	(2, III)	120	95	95.4	
*msh4-E425A*	(2, III)	119	92.6	89	
*msh4-D453A*	(1, IV)	120	93.8	88.2	
*msh4-R456A*	(1, IV)	100	61	40.5	1.9±0.4
*msh4-E461A*	(2, IV)	118	92.4	94.2	
*msh4-Y485A*	(4, IV)	119	94.7	76.7	
*msh4-F491A*	(1, IV)	100	91	47.6	2.5±1.2
*msh4-L493A*	(4, IV)	100	75	43.5	1.6±0.25
*msh4-I495A*	(4, IV)	120	91.7	79.5	
*msh4-N532A*	(1, IV)	118	89.4	64.5	
*msh4-R534A*	(2, IV)	119	91.8	74.3	
*msh4-I542A*	(4, IV)	99	85	59.1	
*msh4-L553A*	(4, IV)	119	95	84.9	
*msh4-G639A*	(4, V)	99	30	42.6	11.6±6.1
*msh4-R676W*	(4, V)	120	89.6	55.6	96±6
*msh4-E732A*	(2, V)	99	93	82.6	
*msh4-H764A*	(2, V)	119	94.5	81.6	
*msh4-D772A*	(2, V)	120	91.3	67.4	
*msh5-E45A*	(3, II)	120	91.5	83.6	62±5.6
*msh5-D76A*	(1, II)	100	88	53.9	1.3±0.25
*msh5-E135A*	(3, II)	179	93.9	89.5	
*msh5-D147A*	(3, II)	120	96.7	87.7	
*msh5-F161A*	(1, II)	119	90.3	74.8	
*msh5-N182A*	(1, II)	120	91	83.2	63±5.1
*msh5-D250A*	(1, II)	99	91	60	6.2±2.2
*msh5-W298A*	(3, III)	120	40.2	30.6	1.4±0.05
*msh5-S416A*	(3, III)	200	90.9	60	3.0±2.1
*msh5-T423A*	(3, III)	120	95.2	78.3	
*msh5-D433A*	(1, IV)	120	47.3	37	1.4±0.05
*msh5-R436A*	(1, IV)	119	50.2	37.6	1.3±0.05
*msh5-Y480A*	(3, IV)	100	67	37.8	2.2±1
*msh5-Y486A*	(1, IV)	120	93.8	62.9	
*msh5-V488A*	(4, IV)	119	39.7	39.6	1.4±0.05
*msh5-I490A*	(4, IV)	120	96	80.1	
*msh5-E495A*	(3, IV)	120	92.3	73.9	
*msh5-D527A*	(1, IV)	116	30.2	34.3	1.9±0.28
*msh5-D532A*	(3, IV)	100	64.5	38.7	3.7±0.0
*msh5-I537A*	(4, IV)	119	87.8	66.1	
*msh5-D539A*	(3, IV)	180	90.4	63.9	89±23.5
*msh5-L548A*	(4, IV)	120	50.2	36.1	
*msh5-G648A*	(4, V)	117	33.3	34	45±1
*msh5-Y661A*	(3, V)	120	45.8	33.6	1.2±0.1
*msh5-D680A*	(3, V)	100	75	38.6	1.4±0.11
*msh5-R685W*	(4, V)	120	36	35.2	46±11.6
*msh5-R837A*	(3, V)	120	93.8	78.7	
*msh5-F876A*	(3, V)	100	94.3	83.6	

Percent spore viability and the genetic map distance (sum of four genetic intervals, *URA3-LEU2*, *LEU2-LYS2*, *LYS2-ADE2*, *ADE2-HIS3*; [24]) from single spores are shown for each of the fifty-seven *msh4* and *msh5* alleles. Amino acid substitutions indicate the wild-type residue, amino acid position, mutation. The different classes indicate; 1: amino acids conserved in Msh4 and Msh5 in five species (*S. cerevisiae*, *A. thaliana*, *C. elegans*, *M. musculus and H. sapiens*) but absent in *S. cerevisiae* Msh2, Msh3, Msh6. 2: amino acid residues conserved in Msh4 only across five species. 3: amino acid residues conserved in MSH5 only across five species. 4: amino acid residues conserved in Msh4 and Msh5 across five species as well as in *S. cerevisiae* Msh2, Msh3 and Msh6. Mutations were also mapped with respect to specific domains in *Taq* MutS. *msh4-G639A* and *msh5-G648A* are analogous to Msh2 ATP binding mutations [55]. *msh4-R676W* and *msh5-R685W* are analogous to Msh2 and Msh6 ATP hydrolysis mutations [53]. Recombination frequencies (recombinant spores/total spores) were multiplied by 100 to obtain genetic map distance in centimorgans (cM). The total number of tetrads dissected (n) for each mutant is shown. Wild-type and *msh5Δ* data are from Argueso *et al.* [24]. Yeast two-hybrid analysis was performed for the indicated *msh4* and *msh5* mutants. β-galactosidase activity (Miller units ± standard deviation) from three independent co-transformants involving the *msh4* and *msh5* mutants and the corresponding wild-type *MSH4* or *MSH5* partner is shown.

### Msh5 appears more sensitive to mutagenesis than Msh4

**Figure 2:**
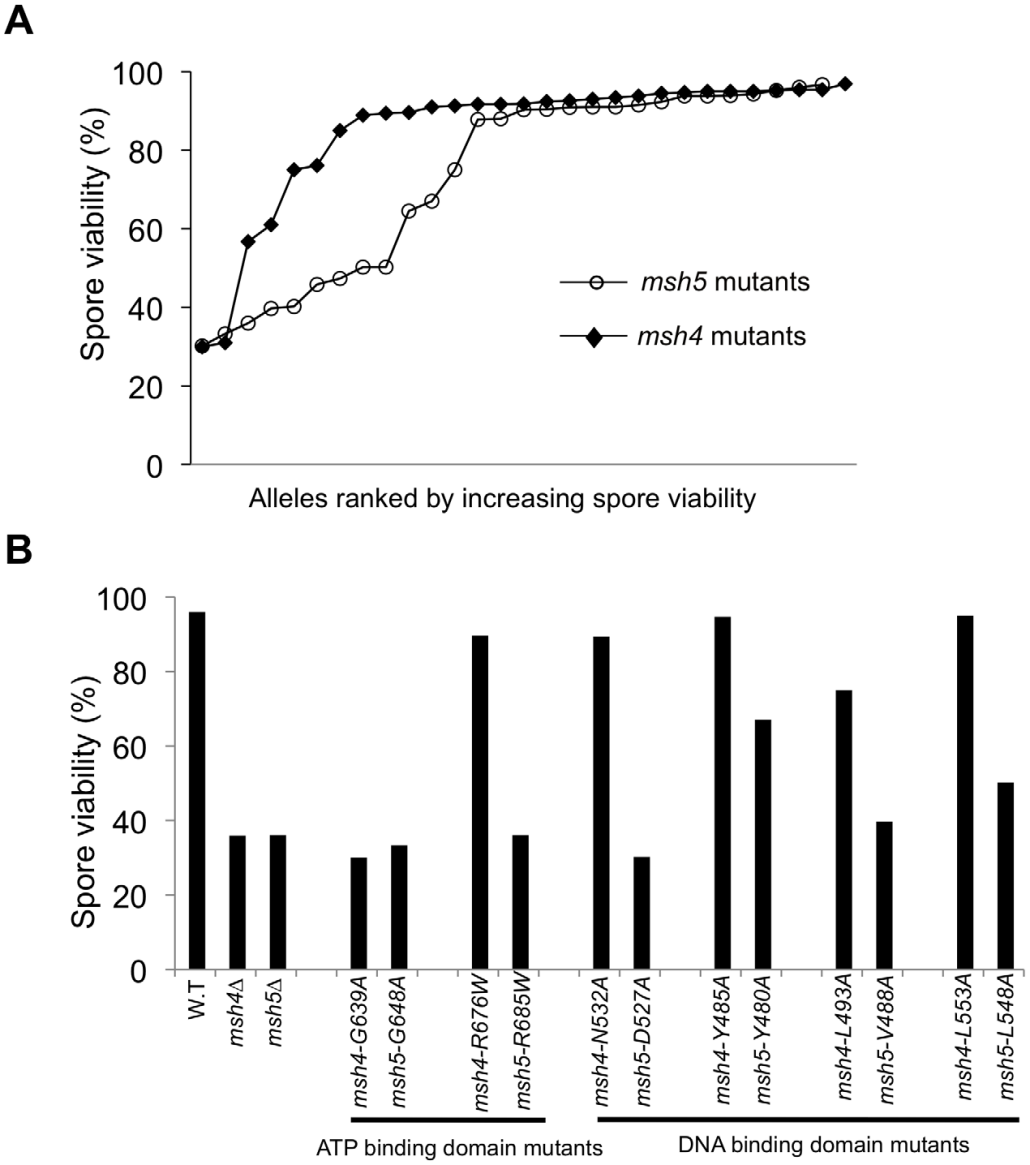
The Msh5 subunit is more sensitive to mutagenesis. (A) Comparison of spore viability of 29 *msh4* and 28 *msh5* mutants in ascending order in the EAY background. (B) Spore viability of conserved pairs of residues in Msh4, Msh5 ATP binding domain and DNA binding domain. *msh4-G639A* and *msh5-G648A* contain mutations analogous to ATP binding mutations in Msh2 while *msh4-R676W* and *msh5-R685W* contain mutations analogous to ATP hydrolysis mutations in Msh2. *msh4-N532A*, *msh4-Y485A*, *msh4-L493A*, *msh4-L553A*, and their matched mutations in Msh5 (*msh5-D527A*, *msh5-Y480A*, *msh5-V488A*, *msh5-L548A*) are conserved within the DNA binding domain (IV). The number of tetrads dissected for each strain is presented in Table 1.

*msh4* and *msh5* alleles were analyzed as heterozygotes over their respective deletion mutations in the SK1 congenic strain EAY1112 [24]. The mutant diploid strains were sporulated and assessed for spore viability and genetic map distances on chromosome XV (Table 1; Figure 1C). The mutations are presented relative to *Thermus aquaticus* MutS domains II, III (linker), IV (DNA binding) and V (ATPase) [35]. The spore viability profiles of *msh4* and *msh5* mutants indicated that the Msh5 subunit was more sensitive to mutagenesis (Figure 2A). A larger proportion of *msh5* mutants showed ≤50% spore viability compared to *msh4* (9 of 28 for *msh5* versus 2 of 29 of *msh4*; p = 0.02, Fisher's exact test). This difference was also seen in an analysis of mutations in domain IV (DNA binding); 5 of 12 *msh5* mutations conferred ≤50% spore viability compared to 0 of 11 *msh4* mutations (p = 0.03, Fisher's exact test).

Five of the eight mutations in homologous positions in Msh4 and Msh5 conferred subunit-specific phenotypes. Both *msh4-G639A* and *msh5-G648A* strains contain mutations (Walker motif A) predicted to disrupt ATP binding; both of these strains displayed null phenotypes [35]–[36], [52]–[55]. In contrast, a predicted ATP hydrolysis mutation in Msh4, *msh4-R676W*, conferred wild-type spore viability but the corresponding mutation in Msh5, *msh5*-*R685W*, conferred a null phenotype (Figure 2B; Table 1). Similar asymmetries between Msh4 and Msh5 were observed at four residues in the DNA binding domain IV (Figure 2B; Table 1). *msh4-N532A*, *msh4-Y485A*, *msh4-L493A*, and *msh4-L553A* had spore viabilities of 89, 95, 75, and 95%, respectively; corresponding mutants *msh5-D527A*, *msh5-Y480A*, *msh5V-488A*, and *msh5-L548A* had significantly lower spore viabilities (30, 67, 40, and 50%, respectively).

Most *msh4* and *msh5* mutants with significant spore viability and/or crossover defects could not form stable Msh4-Msh5 complexes as assessed in the two-hybrid assay (Table 1). The only exceptions were *msh4-E276A* (domain II), *msh4*-*R676W* (ATP hydrolysis), *msh5-D539A* (domain IV), *msh5-G648A* (ATP binding), and *msh5-R685W* (ATP hydrolysis) mutants that displayed poor spore viability or crossover defects but formed stable complexes with a wild-type partner. Inability to form a stable complex in the two-hybrid assay can be explained by the disruption of an interaction domain or a loss in protein stability. Because most mutations were created in highly conserved residues that lie outside of putative interaction domains in Msh proteins [35], [36], [54], a defect in the two-hybrid assay is likely to reflect a disruption of protein structure.

### A threshold level of crossing over is sufficient to ensure spore viability

**Figure 3:**
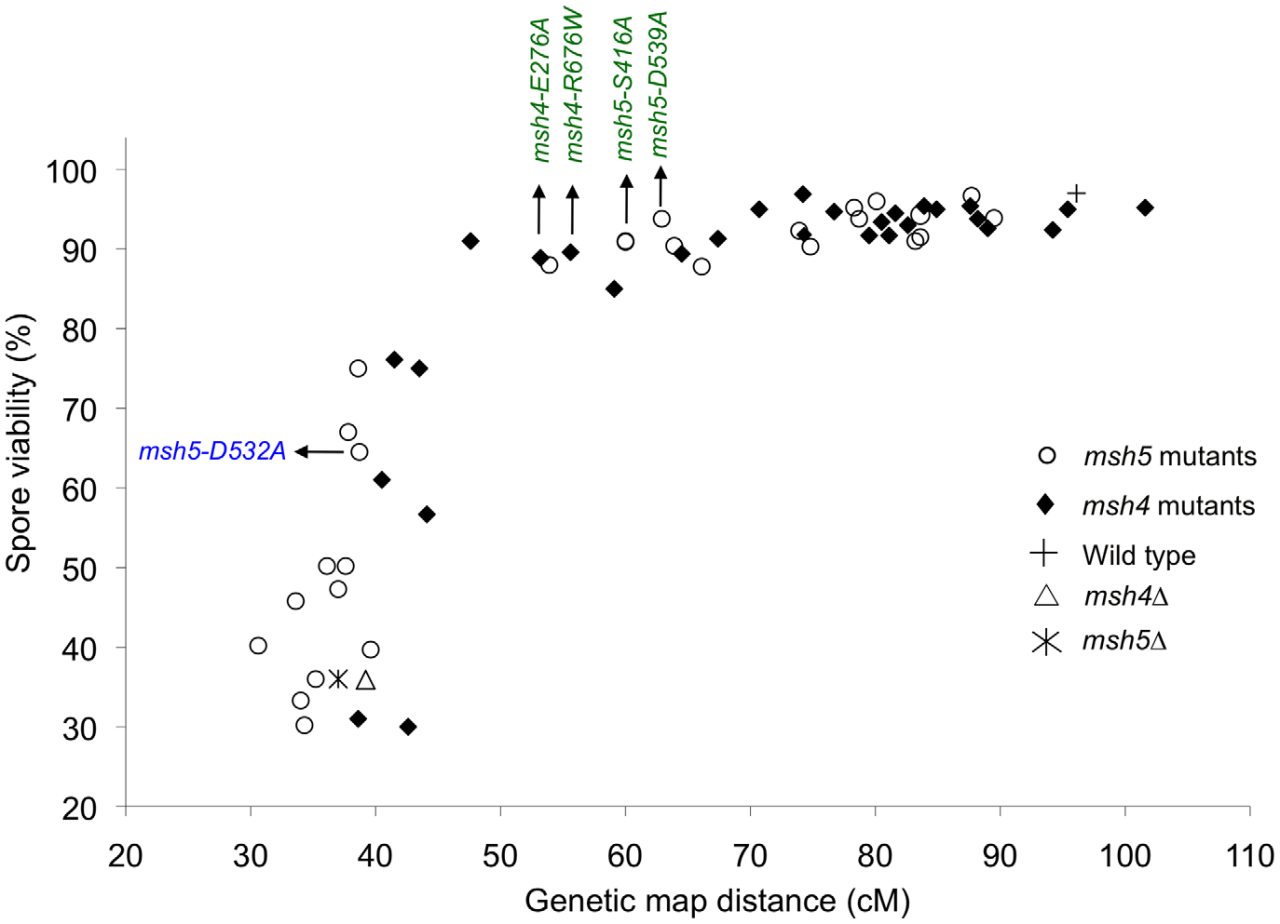
Crossovers can be reduced to a threshold level without affecting spore viability. Plot of spore viability versus genetic map distance on chromosome XV in 57 *msh4*, *msh5* mutants in the EAY strain background. Wild-type, *msh4Δ*, and *msh5Δ* data were also plotted. The *msh4/5-t* (green font) and *msh4/5-bt* (blue font) alleles analyzed in greater depth are shown. Raw data are shown in Table 1.

Spore viability was plotted as a function of genetic map distance for all *msh4* and *msh5* mutants (Figure 3). This plot shows that crossing over could be reduced by up to two-fold on the large chromosome XV without affecting spore viability. *msh4/5* mutations (*msh4/5-t*) near the threshold limit for crossovers included *msh4-E276A*, *msh4-F491A*, *msh4-N532A*, *msh4-R676W*, *msh5-D76A*, *msh5-D250A*, *msh5-S416A*, *msh5-Y486A*, and *msh5-D539A* (Table 1). The phenotypes conferred by these mutations were independent of their ability to disrupt the Msh4-Msh5 complex as measured in the two-hybrid assay (Table 1). A second class of *msh4/5* mutants showed greater than two-fold decreases in crossing over on chromosome XV. This below-threshold class (*msh4/5-bt*; *msh4-Y143A*, *msh4-F194A*, *msh4-R456A*, *msh4-L493A*, *msh5-R436A*, *msh5-Y480A*, *msh5-D532A*, *msh5-L548A*, *msh5-D680A*) showed spore viabilities between 50 and 76%. These mutants were all defective in their ability to form stable Msh4-Msh5 complexes in the two-hybrid assay (Table 1).

### *msh4/5-t* mutants display a preferential loss of crossing over on large chromosomes

**Figure 4:**
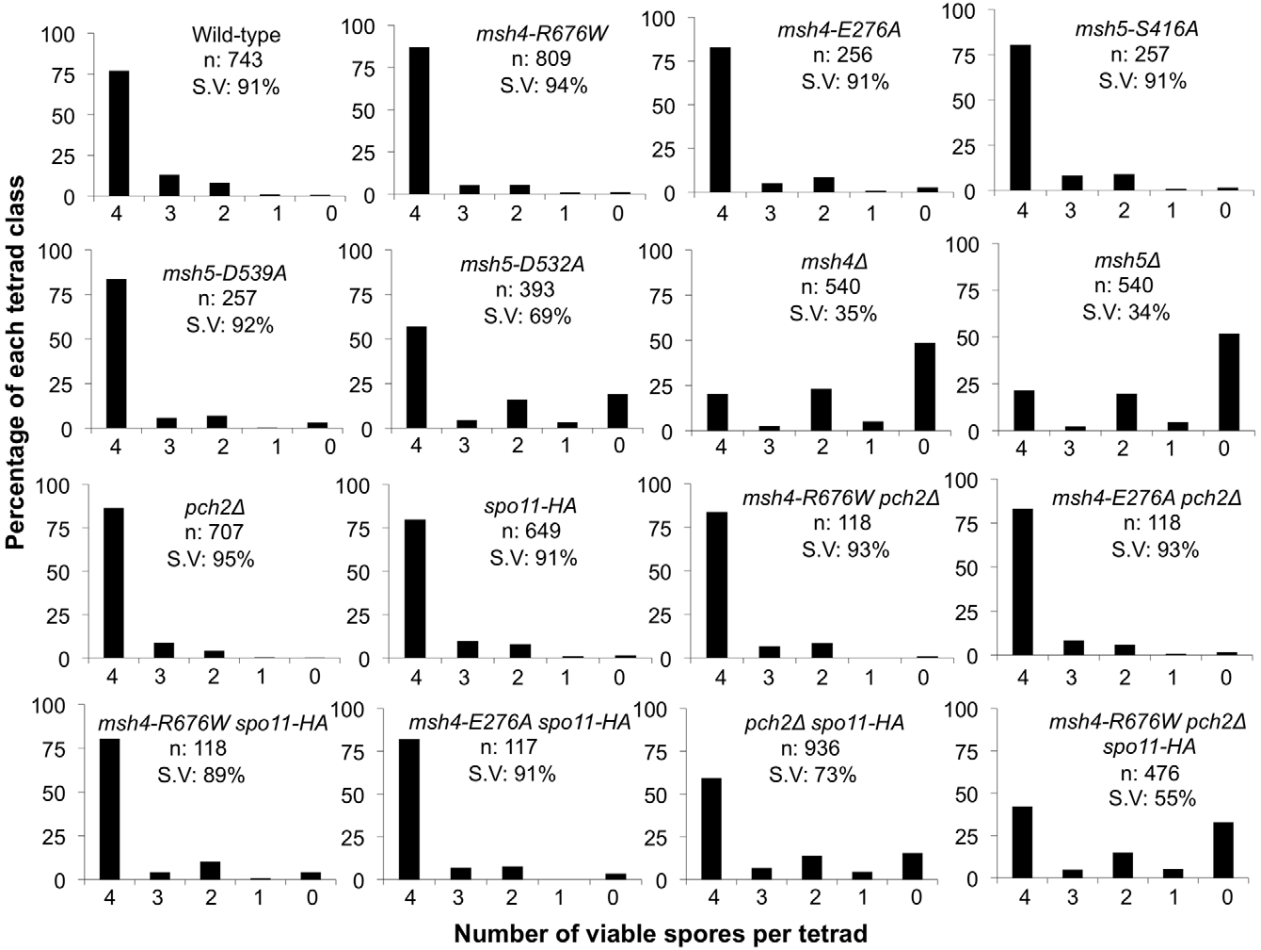
Spore viability profile of wild-type and mutant strains in the NHY942/943 strain background. The vertical axis shows the percentage of each tetrad class and the horizontal axis represents the number of viable spores in a tetrad. n: total number of tetrads dissected, SV: percentage spore viability. Data for wild-type, *pch2Δ*, *spo11-HA* and *pch2Δ spo11-HA* are from Zanders and Alani [21].

**Figure 5:**
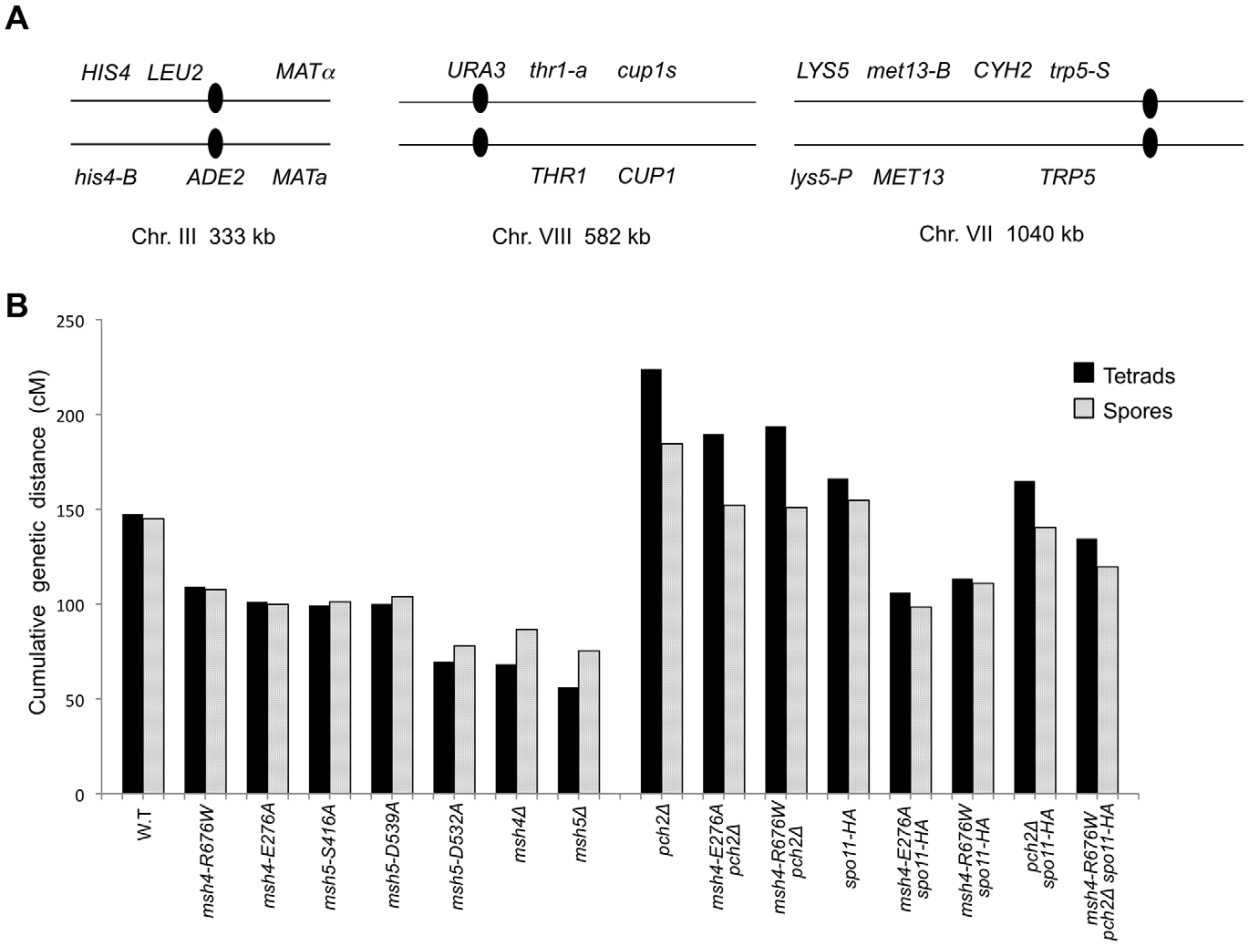
Cumulative genetic map distance in *msh4/5* hypomorphs and double and triple mutations with *pch2Δ* and *spo11-HA*. (A) Location of genetic markers assayed on chromosomes III, VII and VIII in the NHY strain background. Solid circle indicates the centromere. (B) Sum of the genetic map distance (from total spores and complete tetrads) over chromosomes III, VII and VIII in the NHY942/NHY943 strain background. Raw data are shown in Table 2. Data for wild-type, *pch2Δ, spo11-HA*, and *pch2Δ spo11-HA* are from Zanders and Alani [21].

The wild-type spore viability profile for the *msh4/5-t* mutants suggested they were able to properly segregate all sixteen homolog pairs in Meiosis I (Table 1; Figure 3, Figure 4). We further examined the phenotype of a subset of *msh4/5-t* mutants (*msh4-E276A*, *msh4-R676W*, *msh5-S416A*, *msh5-D539A*; all but *msh5-S416A* showed wild-type two-hybrid interactions) in the SK1 isogenic NHY strain background. *msh4* and *msh5* alleles were analyzed as heterozygotes over their respective deletion mutations. The NHY diploid strains allowed us to measure genetic map distances in large (VII), medium (VIII), and small (III) chromosomes (Figure 5A; [23]). Smaller chromosomes have higher map distances per physical distance and weaker interference relative to larger chromosomes ([40], [56], [57] but see [58]). Thus we used this strain set to determine if *msh4/5-t* mutations altered crossover patterns on representative small, medium, and large chromosomes.

All four *msh4/5-t* mutants displayed wild-type spore viability but decreased crossing over (∼1.5-fold for the sum of map distances in three chromosomes; Figure 4, Figure 5B; Table 2). The spore viabilities of wild-type and one *msh4/5-t* mutant, *msh4-R676W*, were unaffected by raising the sporulation temperature to 33°C, a condition shown previously in the SK1 background to cause coordinated defects in the formation of recombination intermediates and crossover products in *msh5Δ* (data not shown; [16]). This observation provides another indication that *msh4/5-t* alleles confer sufficient Msh4-Msh5 function in meiosis. The sum of genetic map distances calculated from tetrads (similar values were obtained from total spores) in wild-type was 147 cM; map distances for *msh4-E276A*, *msh4-R676W*, *msh5-S416A* and *msh5-D539A* were 101, 109, 99, and 100 cM, respectively.

**Table 2:** Genetic map distances and distribution of parental and recombinant progeny in *msh4*, *msh5* mutants in the NHY942/NHY943 strain background.

	Single spores					Tetrads					
Genotype	n	Par.	Rec.	cM	95% C.I	N	PD	TT	NPD	cM	S.E
**Chromosome III**											
*HIS4-LEU2*											
Wild-type	2711	2360	351	12.9	11.7–14.3	572	413	141	2	13.8	1.2
*msh4-R676W*	3041	2763	278	9.1	8.2–10.2	704	562	116	1	9.0	0.8
*msh4-E276A*	933	841	92	9.9	8.1–11.9	212	165	42	0	10.1	1.4
*msh5-S416A*	939	870	69	7.3	5.8–9.2	207	174	26	0	6.5	1.2
*msh5-D539A*	942	875	67	7.1	5.6–8.9	215	182	29	0	6.9	1.2
*msh5-D532A*	1089	1016	73	6.7	5.4–8.3	224	192	24	1	6.9	1.7
*msh4Δ*	760	716	44	5.8	4.3–7.7	110	93	11	0	5.3	1.5
*msh5Δ*	739	708	31	4.2	3.0–5.9	116	102	8	0	3.6	1.2
*pch2Δ*	2691	2302	389	14.5	13.2–15.8	611	421	148	3	14.5	1.3
*spo11-HA*	2371	2144	227	9.6	8.4–10.8	518	409	95	1	10.0	1.0
*pch2Δ spo11-HA*	2715	2454	261	9.6	8.6–10.8	556	437	100	1	9.9	1.0
*msh4-R676W pch2Δ*	440	398	42	9.5	7.1–12.7	99	81	16	0	8.2	1.9
*msh4-E276A pch2Δ*	441	390	51	11.6	8.9–14.9	99	75	19	1	13.2	3.7
*msh4-R676W spo11-HA*	420	398	22	5.2	3.5–7.8	95	83	10	0	5.4	1.6
*msh4-E276A spo11-HA*	426	392	34	8.0	5.7–10.9	96	79	14	0	7.5	1.9
*msh4-R676W spo11- HA pch2Δ*	1040	955	85	8.2	6.7–10.0	201	166	25	1	8.1	1.9
*LEU2-CEN3*											
Wild-type	2711	2527	184	6.8	5.9–7.8	572	488	68	0	6.1	0.7
*msh4-R676W*	3041	2816	225	7.4	6.5–8.4	704	585	93	1	7.3	0.8
*msh4-E276A*	933	876	57	6.1	4.7–7.8	212	182	25	0	6.0	1.1
*msh5-S416A*	939	854	85	9.1	7.4–11.1	207	170	30	0	7.5	1.3
*msh5-D539A*	942	880	62	6.6	5.2–8.3	215	183	28	0	6.6	1.2
*msh5-D532A*	1089	1009	80	7.3	6.0–9.0	224	198	19	0	4.4	1.0
*msh4Δ*	760	678	82	10.8	8.8–13.2	110	96	8	0	3.8	1.3
*msh5Δ*	739	685	54	7.3	5.6–9.4	116	104	6	0	2.7	1.1
*pch2Δ*	2691	2450	241	9.0	7.9–10.1	611	476	96	0	8.4	0.8
*spo11-HA*	2371	2161	210	8.9	7.8–10.1	518	421	84	0	8.3	0.8
*pch2Δ spo11-HA*	2715	2454	261	9.6	8.6–10.8	556	443	93	2	9.8	1.1
*msh4-R676W pch2Δ*	440	406	34	7.7	5.6–10.6	99	83	13	1	9.8	3.5
*msh4-E276A pch2Δ*	441	409	32	7.3	5.2–10.1	99	84	10	1	8.4	3.5
*msh4-R676W spo11-HA*	420	388	32	7.6	5.4–10.6	95	81	12	0	6.5	1.7
*msh4-E276A spo11-HA*	426	403	23	5.4	3.6–8.0	96	86	7	0	3.8	1.4
*msh4-R676W spo11-HA pch2Δ*	1040	950	90	8.7	7.1–10.5	201	168	24	0	6.3	1.2
*CEN3-MAT*											
Wild-type	2711	2309	402	14.8	13.5–16.2	572	395	160	1	14.9	1.0
**Genotype**	**n**	**Par.**	**Rec.**	**cM**	**95% C.I**	**N**	**PD**	**TT**	**NPD**	**cM**	**S.E**
*msh4-R676W*	3041	2629	412	13.5	12.4–14.8	704	500	175	4	14.7	1.2
*msh4-E276A*	933	803	130	13.9	11.9–16.3	212	151	54	2	15.9	2.5
*msh5-S416A*	939	835	104	11.1	9.2–13.2	207	155	45	0	11.3	1.5
*msh5-D539A*	942	807	135	14.3	12.2–16.7	215	154	57	0	13.5	1.5
*msh5-D532A*	1089	1001	88	8.1	6.6–9.8	224	182	35	0	8.1	1.3
*msh4Δ*	760	719	41	5.4	4.0–7.2	110	97	7	0	3.4	1.2
*msh5Δ*	739	716	23	3.1	2.0–4.6	116	104	6	0	2.7	1.1
*pch2Δ*	2691	2317	374	13.9	12.6–15.3	611	418	153	1	13.9	1.1
*spo11-HA*	2371	2084	287	12.1	10.8–13.5	518	388	112	5	14.1	1.6
*pch2Δ spo11-HA*	2715	2533	182	6.7	5.8–7.7	556	472	66	0	6.1	0.7
*msh4-R676W pch2Δ*	440	412	28	6.4	4.4–9.0	99	84	13	0	6.7	1.7
*msh4-E276A pch2Δ*	441	383	58	13.2	10.3–16.6	99	72	20	3	20.0	5.6
*msh4-R676W spo11-HA*	420	389	31	7.4	5.2–10.3	95	79	14	0	7.5	1.9
*msh4-E276A spo11-HA*	426	387	39	9.2	6.8–12.3	96	76	17	0	9.1	2.0
*msh4-R676W spo11-HA pch2Δ*	1040	988	52	5.0	3.8–6.5	201	170	21	1	7.0	1.9
**Chromosome VII**											
*TRP5-CYH2*											
Wild-type	2711	1803	908	33.5	31.7–35.2	572	197	337	9	36.0	1.8
*msh4-R676W*	3041	2379	662	21.8	20.3–23.2	704	378	282	3	22.6	1.2
*msh4-E276A*	933	743	190	20.4	17.9–23.1	212	125	77	2	21.8	2.6
*msh5-S416A*	939	729	210	22.4	19.8–25.1	207	108	84	3	26.2	3.0
*msh5-D539A*	942	736	206	21.9	19.3–24.6	215	115	88	1	23.0	2.2
*msh5-D532A*	1089	881	208	19.1	16.9–21.5	224	136	73	1	18.8	2.1
*msh4Δ*	760	622	138	18.2	15.6–21.1	110	66	30	1	18.6	3.7
*msh5Δ*	739	620	119	16.1	13.6–18.9	116	68	28	0	14.6	2.3
*pch2Δ*	2691	1542	1149	42.7	40.8–44.6	611	129	326	60	66.6	3.9
*spo11-HA*	2371	1492	879	37.1	35.1–39.0	518	149	306	22	45.9	2.8
*pch2Δ spo11-HA*	2715	1699	1016	37.4	35.6–39.3	556	161	311	39	53.3	3.3
*msh4-R676W pch2Δ*	440	257	183	41.6	37.1–46.2	99	22	52	11	69.4	9.9
*msh4-E276A pch2Δ*	441	275	166	37.6	33.2–42.2	99	25	53	6	53.0	7.9
*msh4-R676W spo11-HA*	420	307	113	26.9	22.9–31.3	95	43	46	1	28.9	4.0
*msh4-E276A spo11-HA*	426	340	86	20.2	16.6–24.2	96	57	34	0	18.7	2.5
*msh4-R676W spo11-HA pch2Δ*	1040	730	310	29.8	27.1–32.7	201	78	98	9	41.1	4.7
*CYH2-MET13*											
Wild-type	2711	2451	260	9.6	8.5–10.8	572	442	101	0	9.3	0.8
*msh4-R676W*	3041	2806	235	7.7	6.8–8.7	704	573	89	1	7.2	0.8
*msh4-E276A*	933	873	60	6.4	5.0–8.2	212	178	26	0	6.4	1.2
*msh5-S416A*	939	884	55	5.9	4.5–7.5	207	175	20	0	5.1	1.0
*msh5-D539A*	942	861	81	8.6	7.0–10.6	215	171	33	0	8.1	1.3
*msh5-D532A*	1089	1035	54	5.0	3.8–6.4	224	191	19	0	4.5	1.0
*msh4Δ*	760	715	45	5.9	4.4–7.8	110	89	8	0	4.1	1.4
*msh5Δ*	739	695	44	6.0	4.5–7.9	116	94	1	1	3.6	3.1
*pch2Δ*	2691	2222.5	468.5	17.4	16.0–18.9	611	358	152	5	17.7	1.6
**Genotype**	**n**	**Par.**	**Rec.**	**cM**	**95% C.I**	**N**	**PD**	**TT**	**NPD**	**cM**	**S.E**
*spo11-HA*	2371	2088	283	11.9	10.7–13.3	518	375	102	0	10.7	0.9
*pch2Δ spo11-HA*	2715	2443.5	271.5	10.0	8.9–11.2	556	428	82	1	8.6	1.0
*msh4-R676W pch2Δ*	440	397	43	9.8	7.3–12.9	99	75	9	1	8.8	3.8
*msh4-E276A pch2Δ*	441	390	51	11.6	8.9–14.9	99	70	13	1	11.3	4.0
*msh4-R676W spo11-HA*	420	391	29	6.9	4.8–9.7	95	79	11	0	6.1	1.7
*msh4-E276A spo11-HA*	426	392	34	8.0	5.8–10.9	96	79	10	2	12.1	4.8
*msh4-R676W spo11-HA pch2Δ*	1040	939	101	9.7	8.1–11.7	201	154	30	1	9.7	2.0
*MET13-LYS5*											
Wild-type	2711	2152	559	20.6	19.1–22.2	572	334	205	4	21.1	1.5
*msh4-R676W*	3041	2627	414	13.6	12.4–14.9	704	494	168	1	13.1	1.0
*msh4-E276A*	933	818	115	12.3	10.4–14.6	212	155	49	0	12.0	1.5
*msh5-S416A*	939	815	124	13.2	11.2–15.5	207	147	48	0	12.3	1.5
*msh5-D539A*	942	806	136	14.4	12.3–16.8	215	152	52	0	12.7	1.5
*msh5-D532A*	1089	981	108	9.9	8.3–11.8	224	179	30	1	8.6	1.8
*msh4Δ*	760	656	104	13.7	11.4–16.3	110	76	20	1	13.4	3.6
*msh5Δ*	739	630	109	14.7	12.4–17.5	116	76	19	1	13.0	3.6
*pch2Δ*	2691	1944.5	746.5	27.7	26.1–29.5	611	264	234	17	32.6	2.4
*spo11-HA*	2371	1835	536	22.6	21.0–24.3	518	273	203	1	21.9	1.3
*pch2Δ spo11-HA*	2715	2171.5	543.5	20.0	18.6–21.6	556	340	160	11	22.1	2.1
*msh4-R676W pch2Δ*	440	337	103	23.4	19.7–27.6	99	48	35	2	27.6	5.3
*msh4-E276A pch2Δ*	441	338	103	23.4	19.6–27.5	99	50	32	2	26.2	5.4
*msh4-R676W spo11-HA*	420	349	71	16.9	13.6–20.8	95	64	25	1	17.2	3.9
*msh4-E276A spo11-HA*	426	362	64	15.0	11.9–18.7	96	66	23	2	19.2	5.0
*msh4-R676W spo11-HA pch2Δ*	1040	873	167	16.1	14.0–18.4	201	130	55	0	14.9	1.7
**Chromosome VIII**											
*CEN8-THR1*											
Wild-type	2711	2105	606	22.4	20.8–24.0	572	317	219	2	21.5	1.3
*msh4-R676W*	3041	2557	484	15.9	14.7–17.3	704	467	199	2	15.8	1.1
*msh4-E276W*	933	813	120	12.9	10.9–15.1	212	153	46	0	11.6	1.5
*msh5-S416A*	939	799	140	14.9	12.8–17.3	207	147	54	0	13.4	1.6
*msh5-D539A*	942	828	114	12.1	10.2–14.3	215	155	44	0	11.1	1.5
*msh5-D532A*	1089	973	116	10.7	9.0–12.6	224	180	29	1	8.3	1.8
*msh4Δ*	760	665	95	12.5	10.3–15.0	110	82	15	0	7.7	1.8
*msh5Δ*	739	654	85	11.5	9.4–14.0	116	92	9	0	4.5	1.4
*pch2Δ*	2691	2042	649	24.1	22.5–25.8	611	291	226	7	25.6	1.8
*spo11-HA*	2371	1891	480	20.2	18.7–21.9	518	308	194	3	21.0	1.4
*pch2Δ spo11-HA*	2715	2251	464	17.1	15.7–18.5	556	375	160	4	17.1	1.4
*msh4-R676W pch2Δ*	440	343	97	22	18.4–26.1	99	50	32	1	22.9	4.3
*msh4-E276A pch2Δ*	441	353	88	20	16.5–23.9	99	55	30	1	20.9	4.2
*msh4-R676W spo11-HA*	420	350	70	16.7	13.4–20.5	95	61	31	0	16.8	2.5
*msh4-E276A spo11-HA*	426	379	47	11.0	8.4–14.4	96	75	19	0	10.1	2.1
*msh4-R676W spo11-HA pch2Δ*	1040	856	184	17.7	15.5–20.1	201	129	62	0	16.2	1.7
**Genotype**	**n**	**Par.**	**Rec.**	**cM**	**95% C.I**	**N**	**PD**	**TT**	**NPD**	**cM**	**S.E**
*THR1-CUP1*											
Wild-type	2711	2043	668	24.6	23.0–26.3	572	277	260	1	24.7	1.2
*msh4-R676W*	3041	2475	566	18.6	17.3–20.0	704	432	231	5	19.5	1.3
*msh4-E276A*	933	766	167	17.9	15.6–20.5	212	130	69	0	17.3	1.7
*msh5-S416A*	939	777	162	17.3	15.0–19.8	207	133	68	0	16.9	1.7
*msh5-D539A*	942	764	178	18.9	16.5–21.5	215	127	72	0	18.1	1.7
*msh5-D532A*	1089	967	122	11.2	9.5–13.2	224	173	36	1	10.0	1.9
*msh4Δ*	760	651	109	14.3	12.0–17.0	110	74	23	0	11.9	2.2
*msh5Δ*	739	647	92	12.4	10.3–15.0	116	83	17	1	11.4	3.4
*pch2Δ*	2691	1743	948	35.2	33.4–37.0	611	188	305	31	46.9	3.0
*spo11-HA*	2371	1604	767	32.3	30.5–34.3	518	186	312	7	35.0	1.8
*pch2Δ spo11-HA*	2715	1901	814	30.0	28.3–31.7	556	227	292	20	38.2	2.5
*msh4-R676W pch2Δ*	440	306	134	30.5	26.3–34.9	99	36	43	4	40.4	7.0
*msh4-E276A pch2Δ*	441	320	121	27.4	23.5–31.8	99	43	39	4	36.6	6.8
*msh4-R676W spo11-HA*	420	322	98	23.3	19.5–27.6	95	51	40	1	25.0	4.0
*msh4-E276A spo11-HA*	426	334	92	21.6	18.0–25.7	96	51	42	1	25.5	3.9
*msh4-R676W spo11-HA pch2Δ*	1040	786	254	24.4	21.9–27.1	201	102	83	6	31.2	3.9

All mutants are isogenic derivatives of NHY942/NHY943 (Materials and Methods). For single spores, recombination frequencies (recombinant spores/total spores) were multiplied by 100 to yield genetic map distances (cM). 95% confidence intervals for genetic map distance in the single spores were determined using VassarStats (http://faculty.vassar.edu/lowry/VassarStats.html). For tetrads, genetic distance in centimorgans (cM) was calculated using the RANA software without considering aberrant segregants [24]. The Stahl Laboratory Online Tools website (http://groik.com/stahl/) was used to calculate standard error around the genetic distance for tetrads. n; number of single spores, N; four spore viable tetrads analyzed; Par, parental single spores; Rec, recombinant single spores; S.E; standard error. Wild-type, *pch2Δ*, *spo11-HA* and *pch2Δ spo11-HA* data are from Zanders and Alani [21].

**Figure 6:**
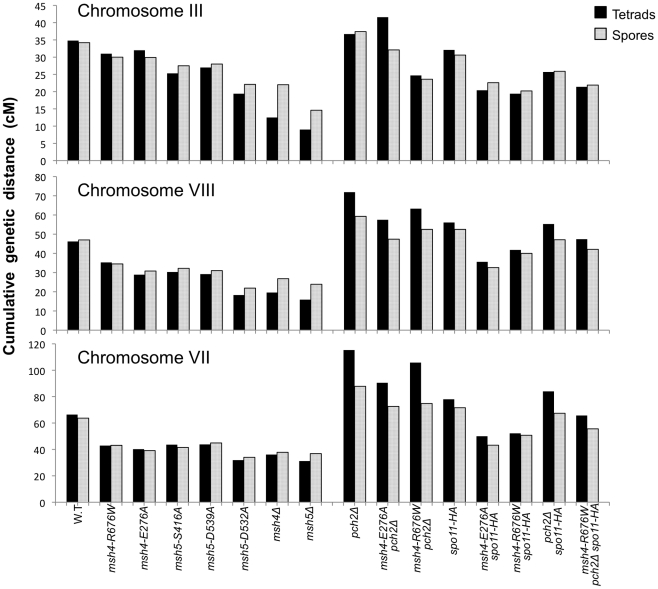
Chromosome size-dependent loss of the meiotic crossover buffer in *msh4/5-t* mutants. Cumulative genetic map distances for chromosomes III, VII, and VIII are shown separately for *msh4/5* hypomorphs as well as their double and triple mutations with *pch2Δ* and *spo11-HA*.

As shown in Figure 6, *msh4/5-t* mutants displayed a chromosome size-dependent loss of crossovers. For three intervals on the smallest chromosome III, the four *msh4/5-t* mutants showed 73 to 92% of wild-type crossover levels (determined from tetrad data). In contrast these mutants showed 63 to 76% of wild-type levels for the two intervals on a medium sized chromosome VIII, and 61 to 66% of wild-type levels for the three intervals on a large chromosome (Chromosome VII). The loss of crossovers on the large chromosome VII approached that seen in *msh4/5Δ* strains. For the *msh4Δ* and *msh5Δ* mutants, the sum of genetic map distances calculated from tetrads was 68 and 56 cM, respectively (2.2 to 2.6-fold drop in crossovers over three chromosomes, Figure 5; Table 2). The values from total spores were 87 and 75 cM for *msh4Δ* and *msh5Δ*, respectively. The differences in map distance calculated by spore and tetrad data were likely due to the high rate of gene conversion seen in *msh4Δ* and *msh5Δ* mutants (see below). Based on tetrad data *msh4Δ* crossovers levels were 36, 42 and 54% of wild-type on chromosomes III, VIII, and VII, respectively. For *msh5Δ* crossover levels were 26, 34 and 47% of wild-type on chromosomes III, VIII, and VII, respectively (Figure 6).

Previously Stahl *et al.* [15] and Abdullah *et al.* [59] reported a greater loss of crossovers on larger chromosomes (VII) compared to smaller ones (III) in *msh4Δ/msh5Δ* mutants. These groups analyzed crossing over in wild-type, *msh4Δ* and *msh5Δ* strains in two intervals (*HIS4*-*LEU2* and *LEU2*-*MAT*) on chromosome III (small) and two (*TRP5*-*CYH2* and *CYH2*-*MET13*) on chromosome VII (large) in the congenic RHB strain background. They found that the crossover defect in *msh4Δ* and *msh5Δ* mutants was stronger on chromosome VII (23% and 27% of wild-type, respectively) compared to chromosome III (39% and 34% of wild type, respectively). We performed our analysis in the NHY SK1 isogenic strain. We do not have a good explanation for why our data differ from the Stahl *et al.* [15] and Abdullah *et al.* [59] studies. One possibility is that genetic mapping information from a limited number of intervals may yield a pattern due to localized recombination effects that is not seen when a larger number of intervals is examined.

We then looked at crossover distribution in a *msh4/5-bt* mutant (*msh5-D532A*). This *msh4/5-bt* mutation conferred similar spore viability levels in the NHY and EAY strain background (65% in EAY vs 69% in NHY; Figure 4). Interestingly, the sum of genetic map distances for chromosomes III, VII, and VIII in *msh5-D532A* (69 cM) was similar to *msh5Δ* (56 cM) and *msh4Δ* (68 cM) (Figure 5); however, *msh5-D532A* showed a preferential retention of crossovers on the small chromosome III. Crossovers in this mutant were 56, 39, and 48 percent of wild-type for chromosomes III, VIII and VII, respectively (determined from tetrads; Table 2; Figure 6).

Gene conversion events were analyzed at eleven marker sites in a subset of *msh4/5* mutants, (*msh4-E276A*, *msh4-R676W*, *msh5-S416A*, *msh5-D532A*, *msh5-D539A*). The frequency of gene conversion in these strains was similar to wild-type (Table 3). As seen previously, *msh4/5Δ* mutants displayed an elevated frequency of gene conversions compared to wild-type [21], [25], [60].

**Table 3:** Percentage of aberrant marker segregation in *msh4*, *msh5* mutants in the NHY942/NHY943 strain background.

Chromosome III	Four- spore viable tetrads	*HIS4*	*LEU2*	*ADE2*	*MATa*	Total
Wild-type	572	2.1	0.3	0.2	0.2	2.8
*msh4-R676W*	704	2.1	1.1	0.0	0.3	3.5
*msh4-E276A*	212	1.9	0.0	0.0	0.5	2.4
*msh5-S416A*	207	1.4	2.4	0.0	0.0	3.8
*msh5-D539A*	215	1.4	0.0	0.0	0.5	1.9
*msh5-D532A*	224	2.7	0.4	0.0	0.0	3.1
*msh4Δ*	110	4.5	0.9	0.0	0.0	5.4
*msh5Δ*	116	3.4	1.7	0.0	0.9	6.0
*pch2Δ*	611	3.8	1.3	0.0	1.3	6.4
*spo11-HA*	518	1.2	0.8	0.0	0.6	2.6
*spo11-HA pch2Δ*	556	2.2	0.9	0.0	0.2	3.3
*msh4-R676W spo11-HA*	95	0.0	2.1	0.0	0.0	2.1
*msh4-E276A spo11-HA*	96	2.1	1.0	0.0	0.0	3.1
*msh4-R676W pch2Δ*	99	1.0	1.0	0.0	0.0	2.0
*msh4-E276A pch2Δ*	99	4.0	0.0	0.0	0.0	4.0
*msh4-R676W spo11-HA pch2Δ*	201	3.5	1.5	0.0	0.0	5.0

Chromosome VII		*LYS5*	*MET13*	*CYH2*	*TRP5*	Total
Wild-type	572	1.6	2.4	0.3	0.7	5.0
*msh4-R676W*	704	1.3	3.0	0.7	1.1	6.1
*msh4-E276A*	212	0.9	1.9	0.0	0.9	3.7
*msh5-S416A*	207	1.9	3.9	0.0	0.0	5.8
*msh5-D539A*	215	0.9	3.3	0.0	0.9	5.1
*msh5-D532A*	224	4.0	1.8	0.4	0.9	7.1
*msh4Δ*	110	3.6	8.2	1.8	0.0	13.6
*msh5Δ*	116	1.7	12.9	2.6	1.7	18.9
*pch2Δ*	611	1.8	11.0	1.8	1.5	16.1
*spo11-HA*	518	0.2	6.8	0.6	0.4	8.0
*spo11-HA pch2Δ*	556	0.4	7.0	0.2	0.7	8.3
*msh4-R676W spo11-HA*	95	0.0	5.3	0.0	0.0	5.3
*msh4-E276A spo11-HA*	96	0.0	3.1	1.0	1.0	5.1
*msh4-R676W pch2Δ*	99	4.0	7.1	2.0	2.0	15.1
*msh4-E276A pch2Δ*	99	4.0	10.1	1.0	0.0	15.1
*msh4-R676W spo11-HA pch2Δ*	201	1.5	5.5	0.0	1.5	8.5

Chromosome VIII		*URA3*	*THR1*	*CUP1*	Total
Wild-type	572	0.2	5.1	0.7	6.0
*msh4-R676W*	704	0.0	4.7	0.6	5.3
*msh4-E276A*	212	0.0	4.7	1.4	6.1
*msh5-S416A*	207	0.0	2.9	0.0	2.9
*msh5-D539A*	215	0.0	6.0	1.4	7.4
*msh5-D532A*	224	0.0	5.8	0.4	6.2
*msh4Δ*	110	0.0	10.0	1.8	11.8
*msh5Δ*	116	0.0	12.1	1.7	13.8
*pch2Δ*	611	0.2	11.9	2.1	14.2
*spo11-HA*	518	0.0	2.1	0.4	2.5
*spo11-HA pch2Δ*	556	0.0	2.9	0.2	3.1
*msh4-R676W spo11-HA*	95	0.0	2.1	1.1	3.2
*msh4-E276A spo11-HA*	96	0.0	2.1	0.0	2.1
*msh4-R676W pch2Δ*	99	1.0	15.2	0.0	16.2
*msh4-E276A pch2Δ*	99	0.0	10.1	3.0	13.1
*msh4-R676W spo11-HA pch2Δ*	201	0.0	4.5	0.5	5.0

Non 2∶2 segregation of markers in *msh4* and *msh5* mutants were identified from four spore viable tetrads using RANA software [24]. All aberrant segregants were 1∶3 or 3∶1 gene conversions except for two 4∶0 events. No post-meiotic segregation events were observed. Gene conversion data for wild-type, *pch2Δ*, *spo11-HA* and *spo11-HA pch2Δ* are from Zanders and Alani [21].

Lastly, crossover interference was analyzed in a representative *msh4/5-t* mutant (*msh4-R676W*) by measuring the coefficient of coincidence (COC, ratio of observed double crossovers to those expected by chance; Table 4; [61]) and the NPD ratio (Table 5; [62]–[63]). Lack of interference yields COC and NPD values of 1 while strong interference yields values significantly less than 1. On the whole crossover interference appeared similar in wild-type and *msh4-R676W*. In COC analysis the *msh4-R676W* mutant showed a lack of interference for two intervals on chromosome III; wild-type showed a lack of interference for only one of these intervals (Table 4). For chromosomes VII and VIII, *msh4-R676W* and wild-type both showed crossover interference at two intervals and the absence of interference at another. NPD ratios, calculated for intervals where at least eight NPD events were expected, were determined using Stahl's “better way” calculator. This method performs a chi square test to determine if there is a significant difference between the observed PD, TT and NPD tetrad classes and those expected by random crossing over. This analysis showed the presence of interference in both wild-type and *msh4-R676W* in three intervals on chromosomes VII and VIII (Table 5).

**Table 4:** Analysis of crossover interference in *msh4-R676W* by coefficient of co-incidence.

Genotype	Four-spore viable tetrads	DCO obs.	DCO exp.	COC	p value	I
**Chromosome III**						
*HIS4-LEU2-CEN3*						
Wild-type	572	5	17.5	0.286	0.004	YES
*msh4-R676W*	704	14	16.2	0.864	0.667	NO
*LEU2-CEN3-MAT*						
Wild-type	572	16	19.7	0.813	0.465	NO
*msh4-R676W*	704	31	24.8	1.251	0.242	NO
**Chromosome VII**						
*TRP5-CYH2-MET13*						
Wild-type	572	57	64.4	0.886	0.363	NO
*msh4-R676W*	704	27	38.7	0.698	0.064	NO
*CYH2-MET13-LYS5*						
Wild-type	572	20	38.9	0.514	0.002	YES
*msh4-R676W*	704	12	22.9	0.523	0.027	YES
**Chromosome VIII**						
*CEN8-THR1-CUP1*						
Wild-type	572	67	107.2	0.625	<0.0001	YES
*msh4-R676W*	704	43	71	0.606	0.0005	YES

The Coefficient of Coincidence (COC) for pairs of adjacent genetic intervals on Chromosomes III, VII and VIII in the NHY strain background was calculated from the ratio of double crossovers observed to that expected using RANA software [24]. Two-tailed p values were calculated using the binomial probabilities calculator with normal distribution. Statistically significant p values (p<0.05) suggest the presence of interference (I) in the genetic interval. Wild-type data are from [21].

**Table 5:** Analysis of crossover interference in *msh4-R676W* by the NPD ratio.

Genotype	Four-spore viable tetrads	NPD Obs.	NPD exp.	Obs./exp.	p value	I
Chromosome VII						
*TRP5-CYH2*						
Wild-type	572	9	33.4	0.269	<0.0001	YES
*msh4-R676W*	704	3	17.0	0.176	0.0001	YES
Chromosome VIII						
*CEN8-THR1*						
Wild-type	572	2	12.5	0.16	0.0007	YES
*msh4-R676W*	704	2	8.15	0.245	0.0186	YES
*THR1-CUP1*						
Wild-type	572	1	17.56	0.056	<0.0001	YES
*msh4-R676W*	704	5	11.63	0.430	0.030	YES

NPD ratio (NPD observed/NPD expected) was calculated from tetrad data presented in Table 2 using the Stahl online laboratory “Better Way” calculator (http://www.molbio.uoregon.edu/~fstahl). p values for the chi square estimate provided by the Better Way calculator were determined using Chi square to p calculator using the VassarStats Web site (http://faculty.vassar.edu/lowry/VassarStats.html) for intervals with a significant number of expected NPD's. Statistically significant p values (*p*<0.05) suggest interference (I) is present in the genetic interval. Wild-type data are from [21].

### High spore viability in *msh4/5-t* mutants requires Pch2-mediated crossover interference

*pch2Δ* mutants display elevated crossing over on medium and large chromosomes, and are defective in crossover interference, yet display wild-type spore viability [21], [64]–[66]. In addition, initial genetic analyses showed that *pch2Δ* mutants displayed an increased ratio of crossovers to non-crossovers [21]. These observations, combined with cytological analyses indicating that Pch2 promotes domainal axis organization in meiosis [66], [67], led Zanders and Alani [21] to propose that Pch2 acts in early steps in crossover control to promote crossover interference at the crossover versus non-crossover decision. To test if *msh4/5-t* mutants showed increased sensitivity to early defects in crossover control, we made double and triple mutant combinations involving the *msh4/5-t*, *spo11-HA*, and *pch2Δ* mutations in the NHY strain background. The *spo11-HA* mutation was examined because strains bearing this allele display a 20% reduction in meiosis specific DSBs but show wild-type levels of crossing over and spore viability due to crossover homeostasis [20]. *pch2Δ spo11-HA* strains, however, display a significant loss in spore viability (73%). One explanation for this phenotype is that when DSBs become limiting, the proper distribution of crossovers becomes even more critical to ensure that every chromosome receives at least one crossover [21],[66].

As shown in Figure 4, Figure 5B, and Table 2, *msh4-R676W spo11-HA* and *msh4-E276A spo11-HA* double mutants displayed wild-type spore viability (89 and 91%, respectively) and cumulative map distances (113 and 106 cM, respectively, from tetrads). These values were similar to those seen in *msh4-R676W* (109 cM) and *msh4-E276A* (101 cM) single mutants. However, compared to *msh4-R676W* and *msh4-E276A* single mutants, *msh4-R676W spo11-HA* and *msh4-E276A spo11-HA* double mutants showed a decrease (∼30%) in crossing over in the small chromosome III that was accompanied by modest increases in crossing over in the medium and large chromosomes (Figure 6; Table 2). We do not have a good explanation for this phenotype; one possibility is that the *spo11* hypomorphs confer mutant phenotypes in addition to lowering DSBs (see Discussion; [21]).

*msh4-R676W pch2Δ* and *msh4-E276A pch2Δ* double mutants also showed wild-type spore viability (93% for both, Figure 4); however the *pch2Δ* mutation conferred an increase in crossing over in *msh4-R676W* and *msh4-E276A* strains that appeared specific to the medium- (VIII) and large-sized (VII) chromosomes (Figure 5B, Figure 6). The cumulative map distances from tetrads in *msh4-R676W pch2Δ* (194 cM) and *msh4-E276A pch2Δ* (190 cM), were higher than wild-type (147 cM) but lower than *pch2Δ* (226 cM; Figure 5B). *pch2Δ msh5Δ* mutants were previously shown to have higher crossover frequencies than the *msh5Δ* mutant [21].

The wild-type spore viability profile seen in *msh4/5-t spo11-HA* suggested that crossover interference and homeostasis can distribute a smaller pool of crossovers to all 16 homolog pairs. In contrast, the wild-type spore viability profile seen in *msh4/5-t pch2Δ* can be explained by an increased number of crossovers compensating for interference defects [21]. Such explanations predict that compromising crossover interference (*pch2Δ*) and limiting DSB's (*spo11-HA*) would decrease spore viability because a random distribution of crossovers will favor large chromosomes (Figure 6; [21]). These effects are likely to be more pronounced in a *msh4/5-t pch2Δ spo11-HA* mutant that is predicted to be compromised for DSB formation, crossover interference, and crossing over. To test this we created the *msh4-R676W pch2Δ spo11-HA* triple mutant and analyzed its phenotype with respect to spore viability, crossover distribution, and chromosome III non-disjunction.

As shown in Figure 4, the *msh4-R676W pch2Δ spo11-HA* triple mutant displayed 55% spore viability, which was lower than *spo11-HA pch2Δ* (72% spore viability). The cumulative crossover level from tetrads for chromosomes III, VII and VIII in this mutant was 135 cM, which was lower than wild-type (147 cM) and *pch2Δ spo11-HA* (165 cM), but significantly higher than *msh4-R676W* (109 cM), which displayed high spore viability (Table 2; Figure 4, Figure 5B). *msh4-R676W pch2Δ spo11-HA* also showed a greater reduction in crossing over on chromosome III compared to *pch2Δ spo11-HA* mutants (Figure 6). Although crossover levels on chromosome III in *msh4-R676W pch2Δ spo11-HA* were similar to *msh4-R676W spo11-HA*, the medium (VIII) and large chromosomes (VII) in *msh4-R676W pch2Δ spo11-HA* showed specific increases in crossing over compared to *msh4-R676W spo11-HA* as predicted by the model (Figure 6, Figure S2). Consistent with this, the triple mutant displayed a spore viability profile indicating a Meiosis I disjunction defect (Figure 4). The triple mutant showed a higher frequency of non-mater two-spore viable tetrads in the triple mutant (12.7%, n = 71 two spore viable tetrads; 1.9% of total tetrads) compared to both *pch2Δ spo11-HA* (6.9%, n = 130; 0.96% of total tetrads) and *msh4-R676W* (6.8%, n = 44; 0.37% of total tetrads). Such tetrads are indicative of nondisjunction of chromosome III because the two viable spores carry both yeast mating types (*MATa* and *MATalpha*). In addition, 82% of the two spore viable tetrads in the triple mutant were sister spores compared to 68% in *pch2Δ spo11-HA* and 50% in *msh4-R676W*. These data are suggestive of non-disjunction of other chromosomes. Together this information is consistent with the triple mutant being unable to distribute at least one crossover between all homolog pairs (see Discussion).

### Functional Msh4-Msh5 is required for complete Zip1 polymerization

**Figure 7:**
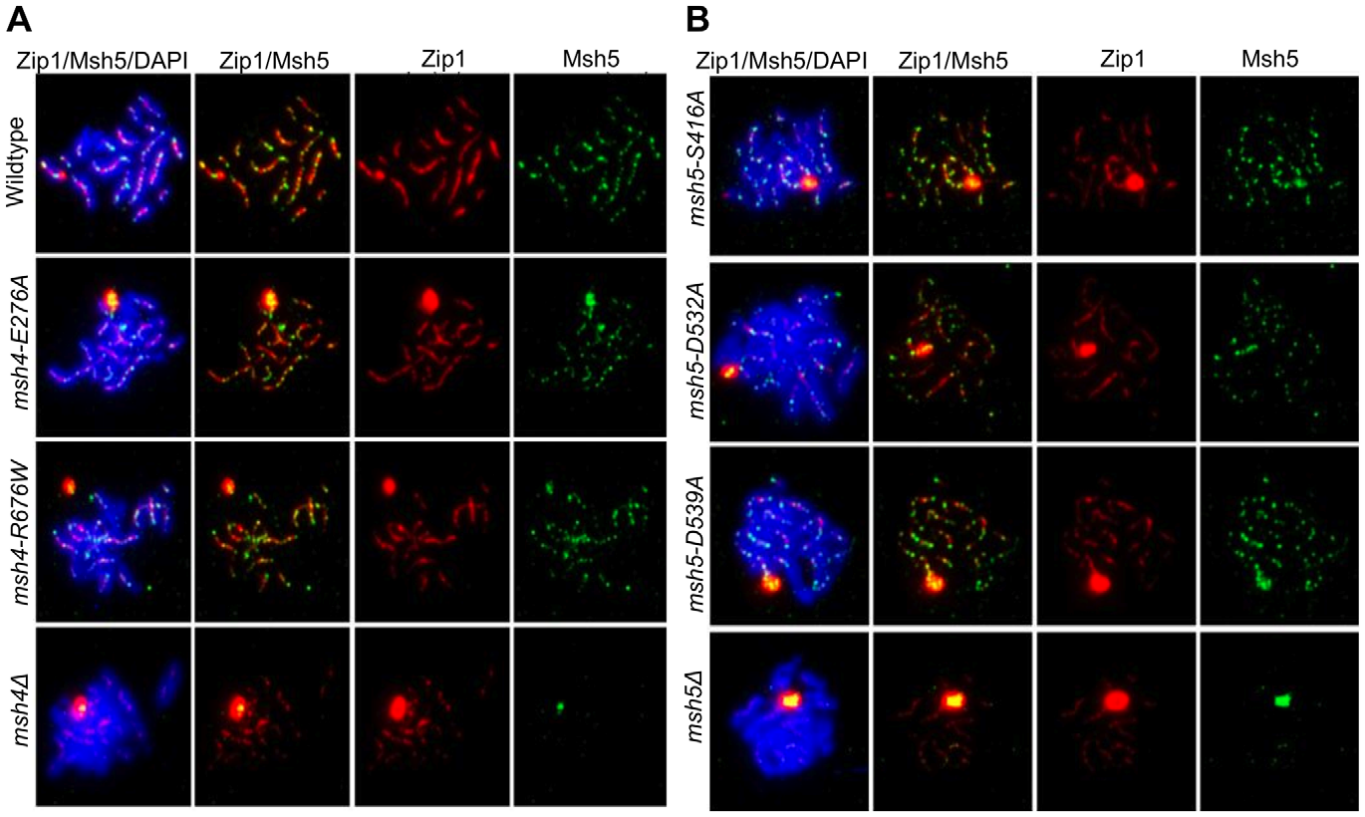
*msh4/5* hypomorphs are defective in Zip1 polymerization. Meiotic chromosome spreads isolated from cells at 4 hr after induction into meiosis were incubated with antibodies to Zip1 and Msh5 and counterstained with DAPI. (A) Localization of Zip1 and Msh5 in wild-type, *msh4-E276A*, *msh4-R676W* and *msh4Δ* mutants. (B) Zip1, Msh5 localization in *msh5-S416A*, *msh5-D532A*, *msh5-D539A* and *msh5Δ* mutants.

*msh4Δ* and *msh5Δ* mutants show strong defects in Zip1 polymerization during synaptonemal complex formation [27], [29]. Our data below indicate that fully functional Msh4-Msh5 is required for complete Zip1 polymerization along homologs. Immunostaining of Msh5 and Zip1 was performed on a subset of the *msh4/5-t* (*msh4-E276A*, *msh4-R676W*, *msh5-S416A*, *msh5-D539A*) and *msh4/5-bt* (*msh5-D532A*) mutants in the NHY strain background four hours after induction into meiosis (Figure 7). The number and distribution of Msh5 foci on meiotic chromosomes for wild-type, *msh4/5-t*, and *msh5-D532A* mutants were similar. The average number of Msh5 foci per nucleus (n = 30) was 122 for wild-type, 120 for *msh5-D532A*, and 130 for *msh5-D539A*. However, all mutants showed a partial defect in Zip1 elongation and accumulated Zip1-specific polycomplexes. This phenotype is reminiscent of that displayed by *spo16* and *zip4* null mutants with the exception that *spo16* and *zip4* null mutants display poor spore viability [29]. One explanation for these observations is that the *msh4/5* mutants present fewer crossover sites to initiate Zip1 polymerization; thus these mutants, while capable of loading Msh4-Msh5 onto meiotic chromosomes, appeared defective in steps required to implement crossing over at designated sites. Thus complete Zip1 polymerization may require feedback from Msh4-Msh5 that is delayed or does not occur in the *msh4/5* mutants.

We also measured by DAPI staining the percent of cells that completed at least Meiosis I (MI/MII) for all of the strains examined by immunofluorescence. As shown in Figure S3, wild-type and one *msh4/5-t* threshold mutant, *msh4-E276A*, displayed similar timing and efficiencies of meiotic divisions. The *msh4Δ*, *msh5Δ*, three *msh4/5-t* mutants (*msh4-R676W*, *msh5-S416A*, *msh5-D539A*), and one *msh4/5-bt* mutant (*msh5-D532A*) all showed about a 1.5 to 2 hr delay relative to wild-type.

## Discussion

We identified *msh4* and *msh5* mutants (*msh4/5-t*) that displayed reduced crossing over in meiosis but maintained crossover interference and wild-type spore viability. The reduction in crossing over seen in *msh4/5-t* mutants appeared more pronounced on large and medium-sized chromosomes that typically receive a greater proportion of Msh4/5-dependent crossovers. *msh4/5-t* mutants also displayed chromosome synapsis defects. These observations and the poor spore viability phenotype of the *msh4-R676W pch2Δ spo11-HA* triple mutant support the idea that baker's yeast form an excessive number of meiotic crossovers and that Pch2-mediated crossover interference is critical for meiotic viability when crossovers become limiting. The *msh4/5-t* alleles, which can be used to titrate crossover levels without reducing spore viability, provide a new tool for investigators interested in identifying factors that regulate crossover control.

### Why does *S. cerevisiae* appear to have an excess of crossovers in meiosis?

*S. cerevisiae* maintains a high level of crossing over, an average of 5.6 per homolog pair [8]–[11], [20]. In most organisms that display crossover interference (*C. elegans*, *A. thaliana*, *Zea mays*, *D. melanogaster*, *Mus musculus* and *Homo sapiens*), the ratio of crossovers in meiosis to homolog pairs is less than or equal to three (reviewed in [68]). Why does *S. cerevisiae* enjoy such a high level of crossing over when a single crossover per homolog pair appears sufficient to promote Meiosis I disjunction [12], [15]? One possibility is that high crossover levels improve fitness by reducing mutational load through the segregation of deleterious alleles [69]. Consistent with this idea are simulation studies suggesting that meiotic crossover rates in *S. cerevisiae* are optimized for mutational robustness [69]. Another possibility is that excess crossovers are needed to ensure crossover formation on small chromosomes [8], [11], [21]. Consistent with the latter explanation is work in yeast showing that a small chromosome (I, 230 KB) has a higher than average recombination rate. Chromosome I also showed a frequency of non-disjunction (0.2–0.4%) that was lower than expected (5%) if it had recombined at the average rate [56], [57], [70]. The enhanced recombination rates on smaller chromosomes in *S. cerevisiae* are likely to result from DSBs that occur at a higher than average density and weak crossover interference [40], [57], [58], [71], [72].

### Models to explain the *msh4/5-t* mutant phenotype

*msh4/5* mutants displayed high spore viability and a higher retention of crossovers on a small chromosome (III) compared to larger chromosomes (VIII, VII and XV). We entertain two models to explain this phenotype. Both of these are based on work showing that Msh4-Msh5 is required to stabilize SEI recombination intermediates and can bind to Holliday junctions [16], [41]. In one model, *msh4/5-t* mutants are defective in converting all SEI and Holliday junction intermediates into crossovers with equal probability. Such a model predicts that crossover interference would not be affected in *msh4/5-t* mutants, and that *msh4/5-t* mutants would show defects in synaptonemal complex formation. Both of these phenotypes were seen in this study. This model predicts that *msh4/5-t* mutants would show high spore viability despite a decrease in crossing over because smaller chromosomes have a higher frequency of crossovers and the number of crossovers in yeast is much greater than the number of chromosomes. A drawback of this model is that it cannot fully explain why *msh4/5* null mutants displayed more severe crossover defects on the smaller chromosome III. Such a pattern is unexpected if crossovers on small chromosomes are present at higher density and occur primarily through a non-interfering pathway [23]. It also cannot explain how *msh4/5-t pch2Δ* mutants make excess crossovers. We cannot rule out the possibility that the small number of intervals examined on chromosome III is not representative of the overall pattern. In the future we would like to test this model further by examining additional intervals on this chromosome as well as on another small chromosome such as chromosome I. In addition, we would like to examine the effect of the *msh4/5-t* mutations on early recombination intermediates such as SEIs.

We considered a second model that proposes a prioritization mechanism for the distribution of crossovers amongst chromosomes. This model is somewhat similar to that proposed by Kaback and colleagues [56], [57]. We suggest that *msh4/5-t* phenotypes reflect a temporal order of crossover designation that favors a crossover on every homolog pair before additional interference-dependent crossovers are made. Such a pattern can be presented within the context of a stress relief model for crossover initiation and distribution. In this model “crossover designation with accompanying interference can be explained by imposition, relief, and redistribution of compression stress and stress relief along chromosome axes” [13]. Crossover initiation on every homolog pair would lead to the release of mechanical stress along the homolog axis of every chromosome. For shorter chromosomes, interference created from stress relief at the crossover initiation site would extend to the end of the chromosome, leading to fewer interfering crossovers as was seen experimentally [57]. For large chromosomes, interference created by stress relief that accompanies obligate crossover designation would prevent additional crossovers until mechanical stresses are re-distributed. We suggest that this redistribution of stress delays additional crossover designations on larger chromosomes. In this model the *msh4/5-t* phenotype can be explained if mutant Msh4-Msh5 complexes can participate in initial stress relief to form an obligate crossover but are defective, perhaps due to stability issues, in subsequent crossover initiations that are subject to interference. This model could explain the synapsis defects seen in *msh4/5-t* mutants if the defect is specific to long chromosomes; a single synapsis initiation site on a small chromosome could be sufficient to allow polymerization along the entire chromosome. This model, however, does not account for why Msh5 focus formation appears wild-type in *msh4/5* mutants. One possibility is that subsequent crossover initiations require functions that occur after Msh4-Msh5 loading onto chromosomes.

The temporal order model outlined above predicts that spore viability would be maintained in *msh4/5-t* mutants due to formation of the obligate crossover and that interference would appear stronger on larger chromosomes. Such an idea is consistent with previous studies in yeast showing that multiple interfering crossovers occur more frequently on large chromosomes and with models that explain the distributions of interfering crossovers seen on different sized chromosomes (e.g. [13], [15], [40], [57], [73]). While we have shown that *msh4/5-t* mutants maintain high spore viability and display crossover interference on large chromosomes (Figure 4; Table 4, Table 5), our data are not robust enough to test whether interference becomes stronger on these chromosomes. A caveat in this model is that *msh4/5-t* mutants display crossover levels on large chromosomes that are higher than wild-type in the *pch2Δ* mutant background. Thus *msh4/5-t* mutants do not appear limited in their ability to form crossovers. One way to explain this observation is that Pch2 acts as a general factor to repress recombination that increases the temporal window over which a mutant Msh4-Msh5 complex must execute crossover decisions. Alleviation of this repression results in increased crossing over in *msh4/5-t pch2Δ* mutants.

Crossovers in *msh4-R676W pch2Δ spo11-HA* triple mutants appear to be randomly distributed, thus leading to more crossing over on larger chromosomes compared to the *msh4-R676W* single mutant, and increased non-disjunction on a small chromosome. Previous studies have suggested that Pch2 is essential for proper meiotic axis organization following crossover designation and that crossover distribution is mediated by changes in meiotic axis organization/assembly (e.g. [13], [67], [74]). We suggest that the triple mutant phenotype can be explained in the second model if the *pch2Δ* mutation disrupts stress/stress relief mechanisms so that crossover designations occur without interference and no crossovers show a temporal delay. In this scenario Pch2 maintains meiotic viability when crossovers are limiting (i.e. *msh4/5-t*, *spo11* hypomorph mutations) because it imposes a delay on additional interfering crossovers. This delay ensures that every homolog pair has received at least one crossover. One way to test this idea in yeast is to perform a genome wide analysis of crossing over in the *msh4/5-t* mutant versus the triple mutant [8], [11].

### Mutations in Msh4 and Msh5 differentially affect function

The Msh family of mismatch repair proteins display asymmetric roles with respect to DNA binding and ATP hydrolysis. In MutS, residues in domain I of subunit A specifically stack with the mismatch while domain IV of subunit B makes non-specific contacts with the DNA backbone [35], [36]. Similarly in MutSα, domain I in Msh6 specifically interacts with the mismatch while domain IV in Msh2 makes non-specific contacts with DNA [54], [75], [76]. Msh subunits also display different affinities for ATP and ADP [77]–[79]. For example in the Msh2-Msh6 mismatch repair complex, Msh6 and Msh2 contain high affinity binding sites for ATP and ADP, respectively [80]. Such asymmetries in ATP binding by Msh subunits are thought to be important to induce coordinated conformational changes in Msh-mismatch DNA complexes that signal downstream repair factors [80]–[84].

Three observations support the presence of asymmetries in Msh4-Msh5 analogous to those seen for the Msh mismatch recognition factors. 1. Snowden *et al.* [85] reported that the Msh4 subunit of human Msh4-Msh5 appears to have reduced ATP binding activity. 2. We identified different spore viability phenotypes for matched sets of *msh4* and *msh5* mutations that map to the ATP and DNA binding domains (Figure 2B). 3. We also found that on the whole, *msh5* mutations conferred more severe meiotic phenotypes than the equivalent *msh4* mutations, though this could indicate different structural organizations for the two proteins rather than asymmetric functions. Msh4-Msh5 binds to both single end invasion and symmetric double Holliday junction substrates [41], [85]. Based on studies performed with Msh and Mlh mismatch repair factors, it is easy to imagine that asymmetric Msh4-Msh5 interactions with its DNA substrate will involve analogous signaling steps that activate downstream factors such as Mlh1-Mlh3. Biochemical analysis of some of the mutant complexes presented in this study can provide evidence to support or refute these ideas.

## Materials and Methods

### Media and yeast strains

*S. cerevisiae* SK1 yeast strains were grown on either yeast extract-peptone-dextrose (YPD) or synthetic complete media at 30°C [86]. When required, geneticin (Invitrogen, San Diego) and nourseothricin (Werner BioAgents, Germany) were added to media at prescribed concentrations [87], [88]. Sporulation medium was prepared as described in Argueso *et al.* [24]. *msh4*, *msh5* mutants were analyzed in either the congenic EAY1108/EAY112 background (“EAY”) described in Argueso *et al.* [24] or the isogenic NHY942/NHY943 background (“NHY”) described in de los Santos *et al.* [23]. 28 *msh5* and 29 *msh4* point mutants were introduced in the EAY1108 background by transformation of EAY1281 and EAY2409 with integration plasmids bearing these mutations using standard techniques [89]. A smaller subset of these *msh4*, *msh5* point mutants were made in the NHY background by transformation of EAY2844 and EAY2848 respectively. Double and triple mutants bearing different combinations of *msh4*, *msh5*, *pch2Δ* and *spo11-HA* were made in the NHY background by crossing single or double mutant strains followed by tetrad dissection. All strains used in this study are listed in Table S1.

### Sequence alignment

Msh4 amino acid sequence from *S. cerevisiae* (YFL003C), *A. thaliana* (NM_117842), *C. elegans* (AF178755), *M. musculus* (BC145838), *H. sapiens* (NM_002440) and Msh5 amino acid sequences from *S. cerevisiae* (YDL154W), *A. thaliana* (EF471448), *C. elegans* (NM_070130), *M. musculus* (NM_013600), *H. sapiens* (BC002498) were aligned using ClustalW software (www.ebi.ac.uk/clustalw) and CLC free workbench. A Msh4, Msh5 consensus sequence was generated using CLC and aligned against *S. cerevisiae* Msh2 (YOL090W), Msh3 (YCR092C), Msh6 (YDR097C) to check if residues conserved across Msh4, Msh5 in all five species are conserved in the other Msh family members.

### Mutagenesis of *MSH4*, *MSH5* genes

The SK1 *MSH4* open reading frame with 600 bp upstream sequence and 400 bp downstream sequence was amplified with *pfu* DNA polymerase and cloned into *pRS416* with a 1.5 kb *KanMX* fragment inserted 90 bp downstream of the *MSH4* stop codon to create the single step integrating plasmid pEAA427. The SK1 *MSH5* open reading frame with 500 bp upstream sequence and 400 bp downstream sequence was similarly amplified with *pfu* DNA polymerase and cloned into *pRS416* with a 1.5 kb *KanMX* fragment inserted 45 bp downstream of the stop codon to create the single step integrating plasmid pEAA424. The *MSH4* and *MSH5* SK1 sequences in these plasmids were confirmed by Sanger DNA sequencing.

pEAA424 and pEAA427 were mutagenized using Quick Change site directed mutagenesis method (Stratagene, La Jolla, CA) to create 28 *msh5* and 29 *msh4* point mutations. The entire open reading frame of *MSH4*, *MSH5* was sequenced to ensure only the desired amino acid change was introduced. Table S1 shows a list of plasmids bearing the *msh4*, *msh5* point mutations.

### Yeast two hybrid analysis

Full length SK1 *MSH4*, *MSH5* and point mutant derivatives were amplified by *pfu* DNA polymerase and cloned into pGAD424 (prey) and target pBTM116 (target) vectors kindly provided by Nancy Hollingsworth. The entire open reading frame of *MSH4*, *MSH5* was checked by DNA sequencing to ensure that no additional mutations were created. The L40 strain [90] was co-transformed with the Prey and Target vectors and expression of the *LACZ* reporter gene was determined by the ortho-nitrophenyl-β-D-galactopyranoside (ONPG) assay [91].

### Tetrad analysis

All *msh4* and *msh5* point mutations integrated into EAY1108 or NHY943 were mated to null strains bearing corresponding *msh4Δ* (EAY2411, EAY background; EAY2843, NHY background) and *msh5Δ* (EAY1280, EAY background; EAY2846, NHY background) alleles. The resulting diploids were sporulated using the zero growth mating protocol [92]. Briefly, the haploid strains were patched together on synthetic complete media for four hours and then spread on sporulation media and incubated for 2 days at 30°C. Tetrads were dissected on synthetic complete media for the EAY background and on YPD media supplemented with amino acids for the NHY background. Spore clones were replica plated onto selective media or minimal drop out plates and incubated overnight. Segregation data were analyzed using the recombination analysis software RANA to determine genetic map distances for tetrads and recombination frequencies for spores [24].

### Cytological analysis of Msh5 and Zip1

Time course, DAPI, and immunostaining analyses of meiotic progression were performed as described using antibodies to Zip1 and Msh5 [29], [93]. Stable SK1 isogenic diploid strains used in the time courses were created by mating the haploid strains shown in parentheses: Wild-type (NHY942×NHY943); *msh4Δ* (EAY2843×EAY2844); *msh4-E276A* (EAY2849×EAY2843), *msh4-R676W* (EAY2851×EAY2843); *msh5Δ* (EAY2846×EAY2848): *msh5-S416A* (EAY2855×EAY2846); *msh5-D539A* (EAY2857×EAY2846); *msh5-D532A* (EAY2785×EAY2846).

## Supporting Information

Figure S1 Clustal W multiple sequence alignment of Msh4 and Msh5 protein sequences from five species. Residues mutated in Msh5 are indicated by solid arrow. Residues mutated in Msh4 are indicated by dotted arrows. Matched pairs of residues mutated in both Msh4 and Msh5 are highlighted in red.
(0.02 MB PDF)

Figure S2 Comparison of the crossover distribution on chromosomes III, VII and VIII in *msh4/5-R676W* versus the *msh4-R676W pch2Δ spo11-HA* triple mutant. Distribution of crossovers from tetrads (left panel) and spores (right panel) across chromosomes III, VII and VIII in the NHY strain background is shown for the *msh4-R676W* and the *msh4-R676W pch2Δ spo11-HA* triple mutant as a percent of wild-type map distance.
(0.23 MB TIF)

Figure S3 Analysis of meiotic divisions in *msh4/5-t* and *msh4/5-bt* cells. Synchronized meiotic cultures of wild-type and *msh4Δ*, *msh5Δ*, *msh4/5-t* (*msh4-E276A*, *msh4-R676W*, *msh5-S416A*, *msh5-D539A) and msh4/5-bt (msh5-D532A)* mutants (strains examined in Figure 7) were analyzed for the completion of at least MI (MI/MII) as measured by DAPI staining. A representative experiment is shown.
(0.26 MB TIF)

Table S1 Strains used in this study. The Plasmid column refers to *MSH4/5::KANMX* and *msh4/5::KANMX* integration vectors used to make the indicated EAY1108 derivative strains.
(0.10 MB DOC)
