# Supplementary material for: Genetic Analysis of Baker's Yeast Msh4-Msh5 Reveals a Threshold Crossover Level for Meiotic Viability
Source: PLoS Genet. 2010 Aug 26;6(8):e1001083. doi: 10.1371/journal.pgen.1001083 (PMC2928781; doi:10.1371/journal.pgen.1001083)
Supplement: Figure S1 — Clustal W multiple sequence alignment of Msh4 and Msh5 protein sequences from five species. Residues mutated in Msh5 are indicated by solid arrow. Residues mutated in Msh4 are indicated by dotted arrows. Matched pairs of residues mutated in both Msh4 and Msh5 are highlighted in red. (0.02 MB PDF) [file pgen.1001083.s001.pdf]

Figure S1

```
CLUSTAL W (1.83) multiple sequence alignment

S. cerevisiae MSH5    ------------------------------------------------------------
A. thaliana   MSH5    ------------------------------------------------------------
C. elegans    MSH5    ---------------------------------------------------------MST 3
H. sapiens    MSH5    ------------------------------------------------------------
M. musculus   MSH5    ------------------------------------------------------------
H. sapiens    MSH4    ------MLRPEISSTSPSAPAVSPS-------------------SGETRSPQGPRYNFGL 35
M. musculus   MSH4    MCCLFLRLRDYSTAHALSLPPCQRCGLQPWSARSHARRTLGVRKAGEMLRQEAASLSSSP 60
A. thaliana   MSH4    ------------------------------------------------------------
C. elegans    MSH4    ------------------------------------------------------------
S. cerevisiae MSH4    -----------------------------------------------MSESNLSSFISTN 13


S. cerevisiae MSH5    --MSHEWLISASETMRSIGNGEGLRDKGAVVANNDGEFNEGDTNREEDSSTIFSFDFDEE 58
A. thaliana   MSH5    ------------------------------------------------MEEMEDTETEPQ 12
C. elegans    MSH5    RWRYYNSKRGNGFRGRGRGRGRGTSLTAVALPRDDNFHKGAQDGAYFKDMPMDPEQFRDE 63
H. sapiens    MSH5    --------MASLGANPRRTPQGP--RPGAASSGFPSP-APVPGPREAEEEEVEEEEELAE 49
M. musculus   MSH5    ---------MAFRATPGRTPPGPGPRSGIPSASFPSPQPPMAGPGGIEEE---DEEEPAE 48
H. sapiens    MSH4    QETPQSR--PSVQVVSASTCPGTSGAAGDRSSSSSSLPCPAPNSRPAQGSYFGNKRAYAE 93
M. musculus   MSH4    RWTPSRRDAPCGRTLASASRPSTEGAMADRSSSSSSSPAPAS----APGSSFGNKRSYAI 116
A. thaliana   MSH4    ------------------------------------------------------------
C. elegans    MSH4    --------------------------------MYSNKSFQRRQRQQVAESRSEEKFSRSL 28
S. cerevisiae MSH4    YFNLRSAANSSNSISKPSTKKSIRNQKSPTNISSWALKKKTLQIAETTWENNEKDSTHSH 73


S. cerevisiae MSH5    IVMCIDFSGGKLGCSILDYHTKTLKAFDQDYVVNKTT----------------------- 95
A. thaliana   MSH5    VYMACIQHGRRVG---------------VSYYDCSVR----------------------- 34
C. elegans    MSH5    TVLSLSFAQGMLGAAYYEQSSQLLKIMNDISEDLEFRFLKRLIDDVKPTLIIANRSQDLE 123
H. sapiens    MSH5    IHLCVLWNSGYLG---------------IAYYDTSDS----------------------- 71
M. musculus   MSH5    IHLCVLWSSGYLG---------------IAYYDTSDS----------------------- 70
H. sapiens    MSH4    NTVASNFTFGASSSSARDTNYPQTLKTPLSTGNPQRSGYKSWTPQVGYSASSSS------ 147
M. musculus   MSH4    HRAASSFPVGTSSSSARDTTYPHTFRTPLSAGNPQRSGHKSWTPQVGYSATSS------- 169
A. thaliana   MSH4    ----------------------------MEDDGGERS----------------------- 9
C. elegans    MSH4    VRLNAQSLLDSSG---------------NNTTTKNVN----------------------- 50
S. cerevisiae MSH4    YLMTGSMASRTATS--------------LSRYSTNASLLG-------------------- 99


S. cerevisiae MSH5    -----ISSHDLIDDADMSSNDISLLLGLLIMEANPTVCLVPARLEDWIFDYIKTKCDEIN 150
A. thaliana   MSH5    -------QLHVLEFWEEDCSDFTLINMVKYQAKPSIIYASTKSEE--SFIAALQQNDGTD 85
C. elegans    MSH5    FIKFLTTRYDPQEKIYEDGTTEEGTSEDTVPTWDSSLAYSTDETTAEKEEKEEDEDDDDE 183
H. sapiens    MSH5    ------TIHFMPDAPDHESLKLLQRVLDEINPQSVVTSAKQDENMTRFLGKLASQEHREP 125
M. musculus   MSH5    ------TIHFMPDAPDHESLKLLQRVLDEINPQSVVTSAKQDEAMTRFLGKLASEEHREP 124
H. sapiens    MSH4    -AISAHSPSVIVAVVEGRGLARGEIGMASIDLKNPQIILSQFADN-TTYAKVITKLKILS 205
M. musculus   MSH4    -AVSAHAPSVIVAVVEGRGLARGEIGMASIDLKSPQIMLSQFADN-TTYAKVITKLQVLS 227
A. thaliana   MSH4    --------SFVAGLIENR---AKEVGMAAFDLRSASLHLSQYIETSSSYQNTKTLLRFYD 58
C. elegans    MSH4    -------SDVVIVVMEGRGSCEGHIGIALHDTCFPEIHLCEFVDS-REYTTLKTMINVHE 102
S. cerevisiae MSH4    ----PSIDCVLCCIYEVPRDISTRIGLCIINCNTGQMYLSDFMDS-QIYIRVVHKLQIYQ 154
```

```
S. cerevisiae MSH5    CRLELQPIKRFKKWDLLQSLQLRGHDNQTILNDILSNSKFTTTVTLGTVGCILANHEQLG 210
A. thaliana   MSH5    ETTMVKLVKSSTFSYEQAWHRLVYLRVTGMDDGLNIKERICYLSSMMDVGSEVQVRVSGG 145
C. elegans    MSH5    GLPAKLNKLPNNFFRMSRAIERLKAMAGSHDSSMTEEDKYIIIKMRFDIEAVNMIRSFGA 243
H. sapiens    MSH5    KRPEIIFLPSVDFGLEISKQRLLSGNYSFIPDAMTATEKILFLSSIIPFDCLLTVRALGG 185
M. musculus   MSH5    KGPEIILLPSVDFGPEISKQRLLSGNYSFISDSMTATEKILFLSSIIPFDCVLTVRALGG 184
H. sapiens    MSH4    PLEIIMSNTACAVGNSTKLFTLITENFKNVNFTTIQRKYFNETKGLEYIEQLCIAEFSTV 265
M. musculus   MSH4    PLEIIMSNTACVVGNSTKLFTLITENFKNVNFTTVQRKYFNETKGLEYIEQLCIAEFSSV 287
A. thaliana   MSH4    PSVIIVPPNKLAADGMVGVSELVDRCYSTVRKVVFARGCFDDTKGAVLIQNLAAEEPLAL 118
C. elegans    MSH4    AFDIVIQNGNEERGSTKLLGEALMTAFPEASLQSISSKYFNSERGERQLQSLMNAEVSTV 162
S. cerevisiae MSH4    PTEILIPSSSLAPTVSKLATMIKFNVAETVKIEEGSRKCFNSQDGLAAITKYLMDDTKKD 214

S. cerevisiae MSH5    EYNDSTAS----SNMVTGRLVQNAFEDVIHGIRYIDIRDRMVLDENTISALHIFPTAHKL 266
A. thaliana   MSH5    LLAILESERIVETLEQNESG---SASIAIDSVMEVPLNKFLKLDAAAHEALQIFQTDKHP 202
C. elegans    MSH5    LLLFLDET---RMGVTDDPL---SVTSPIKSIKTFTLGNLVEIDFNTIQALDILPKETEN 297
H. sapiens    MSH5    LLKFLGR---RRIGVELEDY---NVSVPILGFKKFMLTHLVNIDQDTYSVLQIFKSESHP 239
M. musculus   MSH5    LLKFLSR---RRIGVELEDY---DVGVPILGFKKFVLTHLVSIDQDTYSVLQIFKSESHP 238
H. sapiens    MSH4    LMEVQSK---YYCLAAVAAL---LKYVEFIQNSVYAPKSLKICFQGSEQTAMIDSSSAQN 319
M. musculus   MSH4    LMEVQSR---YYCLAAAAAL---LKYVEFIQNSVYAPKSLKIYFQGSEQTAMIDSSSAQN 341
A. thaliana   MSH4    GLDTYYKQ-HYLSLAAAAAT---IKWIEAEKGVIVTNHSLTVTFNGSFDHMNIDATSVEN 174
C. elegans    MSH4    SEGCLRR---TLALGALAVL---LKYIHETRCVFFRVKSLRIKEMGVNDTCMIDFVSWES 216
S. cerevisiae MSH4    LKIEEIIDKTFALCAASAAISYMEEIISKSSRNLNAFRKLRIQFEGTENTMLIDSKTVRG 274

S. cerevisiae MSH5    GHDKMMRN----------GFFSVFELFNQVSSDYARRILKSWLINPLTNKKRIETRYSII 316
A. thaliana   MSH5    SHMGIGRA---------KEGFSVFGMMNKCATPMGRRLLRSWFMRPILDLEVLDRRLNAI 253
C. elegans    MSH5    KKTFGQGR-------------SLYQLMDKCRSTVGKKCLRKWFRNPTTDRDDLVSRQKCV 344
H. sapiens    MSH5    SVYKVASG--------LKEGLSLFGILNRCHCKWGEKLLRLWFTRPTHDLGELSSRLDVI 291
M. musculus   MSH5    SVYKVASG--------LKEGLSLFGILNRCRCKWGQKLLRLWFTRPTRELRELNSRLDVI 290
H. sapiens    MSH4    LELLINNQ-------DYRNNHTLFGVLNYTKTPGGSRRLRSNILEPLVDIETINMRLDCV 372
M. musculus   MSH4    LELLVNNQ-------DYRSNHTLFGVLNYTKTAGGSRRLRSNILEPLVDVETISMRLDCV 394
A. thaliana   MSH4    LELIDPFHNALL--GTSNKKRSLFQMFKTTKTAGGTRLLRANLLQPLKDIETINTRLDCL 232
C. elegans    MSH4    LEIVDADDASKARKFQMKQKRTLMSVLNHTVTTNGYRLLRSSVLQPSTDVYLIQSRQEAI 276
S. cerevisiae MSH4    LELVENKLD--------KNGISLWKFLDTTSTKMGQRSLRNSILQPLTDRGSIEMRLEAL 326

S. cerevisiae MSH5    RTLLDKQNAIIFSDLSQSIKRCPDAFGFINQLRSGKST---------------LGTWSKV 361
A. thaliana   MSH5    SFFIS--SVELMASLRETLKSVKDISHLLKKFNSPTSLCTS------NDWTAFLKSISAL 305
C. elegans    MSH5    HYFKQ--DWN--AEVTAKLSSILGRVKALNSVFQKFQS--------------GTAQLIHW 386
H. sapiens    MSH5    QFFLL--PQN--LDMAQMLHRLLGHIKNVPLILKRMKL--------------SHTKVSDW 333
M. musculus   MSH5    QFFLM--PQN--LDMAQMLHRLLSHIKNVPLILKRMKL--------------SHTKVSDW 332
H. sapiens    MSH4    QELLQ--DEELFFGLQSVISRF-LDTEQLLSVLVQIPK--------QDTVNAAESKITNL 421
M. musculus   MSH4    QELLQ--DEELFFGLQSVISRF-LDTEQLLSVLVQIPK--------QDTVNAAESKITNL 443
A. thaliana   MSH4    DELMS--NEQLFFGLSQVLRKFPKETDRVLCHFCFKPKKVTEAVIGFENTRKSQNMISSI 290
C. elegans    MSH4    EELIG--KPQLKDKLRRTLSRAHELDRVIAMCIQTSTS---------WTVRESEAKINQI 325
S. cerevisiae MSH4    EELKA--NDDLLQKLRLEMKSLPDLDKLFSRLLCINHS-----------AIKPDQRINYV 373
```

```
S. cerevisiae MSH5    ASFLEKGIAIFQLVSSLKLGSDEANILHDIKNKVDISALKECLRKVETVIDFDTSRDTKT 421
A. thaliana   MSH5    LHVNKIFEVGVSESLREHMRRFNLDIIEKAGLCIST-ELDYVYELVIGVIDVTRSKERGY 364
C. elegans    MSH5    ECFVSTVNALVEILNIIRQTPISKEFPVESDLLREV---SEIAVIAGSIINFAESKIQGR 443
H. sapiens    MSH5    QVLYKTVYSALGLRDACRSLPQSIQLFRDIAQEFSD-DLHHIASLIGKVVDFEGSLAENR 392
M. musculus   MSH5    QVLYKTVYSALGLRDACRSLPQSIQLFQDIAQEFSD-DLHHIASLIGKVVDFEESLAENR 391
H. sapiens    MSH4    IYLKHTLELVDPLKIAMKNCNTPLLR-AYYGSLEDK-RFGIILEKIKTVINDDARYMKGC 479
M. musculus   MSH4    IYLKHTLELVEPLKVTLKNCSTPLLR-AYYGSLEDH-RFGLILDKIKTVINDDARYMKGC 501
A. thaliana   MSH4    ILLKTALDALPILAKVLKDAKCFLLANVYKSVCEND-RYASIRKKIGEVIDDDVLHARVP 349
C. elegans    MSH4    IKLMHTLKVIQGIRTLLHSAKMKSNILIEKTEFLKDPRFDQIMNILVEKVDDSLLDGKKN 385
S. cerevisiae MSH4    LLLKETLQSVKSLKDALNDQLIQSRLISETKKIFNNDAIMEIEKLINSCINEDCVWASSA 433

S. cerevisiae MSH5    LTINTG-VDNRLDECRNIYNHLEGILLDVARETQIFLLNTMPQEDCKTTKSLEKLVNAVY 480
A. thaliana   MSH5    QTLVKEGFCAELDELRQIYEELPEFLQEVSAMELEHFPHLHKEK--LP-------PCIVY 415
C. elegans    MSH5    VTVMNG-IDEELDEIRDTYENMPMVLTAIAKQEEARLGLPPYSN-----------VACVY 491
H. sapiens    MSH5    FTVLPN-IDPEIDEKKRRLMGLPSFLTEVARKELENLDSR------IPS------CSVIY 439
M. musculus   MSH5    FTVLPN-IDPDIDAKKRRLIGLPSFLTEVAQKELENLDSR------IPS------CSVIY 438
H. sapiens    MSH4    LNMRTQ-KCYAVRSNINEFLDIARRTYTEIVDDIAGMISQLGEKYSLP-------LRTSF 531
M. musculus   MSH4    LNMRTQ-KCYAVRSNISEFLDIARRTYTEIVDDIAGMIAQLAEKYSLP-------LRTSF 553
A. thaliana   MSH4    FVARTQ-QCFALKAGIDGFLDIARRTFCDTSEAIHNLASKYREEFNLPN------LKLPF 402
C. elegans    MSH4    SLHLQNTKCYAIRHFVAVQLDLARQTYEEIIRNVEETGAREIAEYFHGNS----SVRLSF 441
S. cerevisiae MSH4    IQLLNQ-RSYAVKSDSNGLLDVSRQIYKEVKEEFFREVEDLTAKNKIN-------LDHNY 485

S. cerevisiae MSH5    IPQLGYLVTIS-----VLMEPLLDGIPNLQWEEIFRSSENIYFKNGRVLELDETYGDIYG 535
A. thaliana   MSH5    IQQIGYLMCIFGEKLDETALNRLTEFEFAFSDMDGETQR-FFYHTSKTRELDNLLGDIYH 474
C. elegans    MSH5    IPLVGFVLSVP---------RDYGVESQPDMTLLYSTHEDLRVRNATTSRLDDEFGDILM 542
H. sapiens    MSH5    IPLIGFLLSIP----RLPSMVEASDFEINGLDFMFLSEEKLHYRSARTKELDALLGDLHC 495
M. musculus   MSH5    IPLIGFLLSIP----RLPFMVEASDFEIEGLDFMFLSEDKLHYRSARTKELDTLLGDLHC 494
H. sapiens    MSH4    SSARGFFIQMT----TDCIALPSDQLPSEFIKISKVKNS-YSFTSADLIKMNERCQESLR 586
M. musculus   MSH4    SSSRGFFIQMT----TDCAALSSDQLPSEFIKISKVKNS-YSFTSADLIKMNERCQESLR 608
A. thaliana   MSH4    NNRQGFFFRIP----QKEVQG---KLPNKFTQVVKHGKN-IHCSSLELASLNVRNKSAAG 454
C. elegans    MSH4    SQSRGFHYTFV------TRQAESVTIPRYFLDVFRNRTT-VTFNSRKVIAYNDRLEQVVA 494
S. cerevisiae MSH4    DSARGFYLRIK----RQEFTDDVATLPDVFISRTIKKNY-IECTTLNIIKKNARLKEVME 540

S. cerevisiae MSH5    AISDFEIEILFSLQEQILRRKTQLTAYNILLSELEILLSFAQVSAERN----YAEPQLVE 591
A. thaliana   MSH5    KILDMERAIIRDLLSHTLLFSAHLLKAVNFVAELDCILSLACVAHQNN----YVRPVLTV 530
C. elegans    MSH5    RLIDSQTAIILTLKTRVMKKKRSIIKLLSIASRIDVLISFGLIAAQNG----WNCPALVD 598
H. sapiens    MSH5    EIRDQETLLMYQLQCQVLARAAVLTRVLDLASRLDVLLALASAARDYG----YSRPRYSP 551
M. musculus   MSH5    EIRDQETLLMYQLQCQVLARASVLTRVLDLASRLDVLLALASAARDYG----YSRPHYSP 550
H. sapiens    MSH4    EIYHMTYMIVCKLLSEIYEHIHCLYKLSDTVSMLDMLLS-FAHACTLS---DYVRPEFTD 642
M. musculus   MSH4    EIYHMTYMIVCKLLSEIYEHIHCLYKLSDTVSMLDMLLS-FAHACTLS---DYVRPEFTD 664
A. thaliana   MSH4    ECFIRTETCLEALMDAIREDISALTLLAEVLCLLDMIVNSFAHTISTKPVDRYSRPELTD 514
C. elegans    MSH4    EMFLASDVIVCDMIEEMQPMIPVLYYAMDALSSIDFLCGLATYSDLRD----TCKPTFGP 550
S. cerevisiae MSH4    EILLLSEETVDELLDKIATHISELFMIAEAVAILDLVCSFTYNLKENN----YTIPIFTN 596
```

```
S. cerevisiae MSH5    DECILEIINGRHALYETFLDNYIPNSTMIDGGLFSELSWCEQNKGRIIVVTGANASGKSV 651
A. thaliana   MSH5    ES-LLDIRNGRHVLQEMAVDTFIPNDTEIN------------DNGRIHIITGPNYSGKSI 577
C. elegans    MSH5    EPVIEAVELYHPISVLVVKKSFVPNQVSSGR-----------DGIKASIITGPNACGKSV 647
H. sapiens    MSH5    QVLGVRIQNGRHPLMELCARTFVPNSTECGG-----------DKGRVKVITGPNSSGKSI 600
M. musculus   MSH5    CIHGVRIRNGRHPLMELCARTFVPNSTDCGG-----------DQGRVKVITGPNSSGKSI 599
H. sapiens    MSH4    T---LAIKQGWHPILEKISAEKPIANNTYVT-----------EGSNFLIITGPNMSGKST 688
M. musculus   MSH4    T---LAIKQGWHPILEKISAEKPVANNTYIT-----------EGSNVLIITGPNMSGKST 710
A. thaliana   MSH4    SGP-LAIDAGRHPILESIHND-FVSNSIFMS-----------EATNMLVVMGPNMSGKST 561
C. elegans    MSH4    S---FSISQGRHPILDWDDSEKTITNDTCLT-----------RDRRFGIITGPNMAGKST 596
S. cerevisiae MSH4    N---LLIRDSRHPLLEKVLKNFVPNTISSTK-----------HSSSLQIITGCNMSGKSV 642

S. cerevisiae MSH5    YLTQNGLIVYLAQIGCFVPAERARIGIADKILTRIRTQETVYKTQSSFLLDSQ-QMAKSL 710
A. thaliana   MSH5    YVKQVALIVFLSHIGSFVPADAATVGLTDRIFCAMG-SKFMTAEQSTFMIDLH-QVGMML 635
C. elegans    MSH5    YMKSIGIMVFLSHIGSFVPARHAKIGIVDRIVTRMFTVDSVLDGMSTFAKDVE-QVALAL 706
H. sapiens    MSH5    YLKQVGLITFMALVGSFVPAEEAEIGAVDAIFTRIHSCESISLGLSTFMIDLNQQVAKAV 660
M. musculus   MSH5    YLKQVGLITFMALVGSFVPAEEAEIGVIDAIFTRIHSCESISLGLSTFMIDLN-QVAKAV 658
H. sapiens    MSH4    YLKQIALCQIMAQIGSYVPAEYSSFRIAKQIFTRISTDDDIETNSSTFMKEMK-EIAYIL 747
M. musculus   MSH4    YLKQIALCQIMAQIGSYVPAEYASFRIAAQIFTRISTDDDIETNSSTFMKEMK-EIAYIL 769
A. thaliana   MSH4    YLQQVCLVVILAQIGCYVPARFATIRVVDRIFTRMGTMDNLESNSSTFMTEMR-ETAFIM 620
C. elegans    MSH4    YLKQTAQLAIMAQIGCFIPANYASLPIFNRIFSRMGHNDELIRNKSAFASEMS-DAAAIV 655
S. cerevisiae MSH4    YLKQVALICIMAQMGSGIPALYGSFPVFKRLHARVC-NDSMELTSSNFGFEMK-EMAYFL 700

S. cerevisiae MSH5    SLATEKSLILIDEYGKGTDILDGPSLFGSIMLNMSKSEK--CPRIIACTHFHELFNENVL 768
A. thaliana   MSH5    RQATSRSLCLLDEFGKGTLTEDGIGLLGGTISHFATCAEP--PRVVVCTHLTELLNESCL 693
C. elegans    MSH5    RKATGNSLVIIDEFGKGTMTEVGLSLLASVMTYWMNRGADRCPHIFLSSHFHALPNYIPL 766
H. sapiens    MSH5    NNATAQSLVLIDEFGKGTNTVDGLALLAAVLRHWLARGPT-CPHIFVATNFLSLVQLQLL 719
M. musculus   MSH5    NNATEHSLVLIDEFGKGTNSVDGLALLAAVLRHWLALGPS-CPHVFVATNFLSLVQLQLL 717
H. sapiens    MSH4    HNANDKSLILIDELGRGTNTEEGIGICYAVCEYLLSLK----AFTLFATHFLELCHIDAL 803
M. musculus   MSH4    HNANDKSLILIDELGRGTNTEEGIGISYAVCEHLLSIK----AFTLFTTHFLELCHLDAL 825
A. thaliana   MSH4    QNVTNRSLIVMDELGRATSSSDGLAMAWSCCEYLLSLK----AYTVFATHMDSLAELATI 676
C. elegans    MSH4    QYADKNSLVVLDELARSTSTEEGIAITYAICEKVLKLQ----SYTFLATHFLDIAALANY 711
S. cerevisiae MSH4    DDINTETLLILDELGRGSSIADGFCVSLAVTEHLLRTEAT----VFLSTHFQDIPKIMSK 756

S. cerevisiae MSH5    TENIKGIKHYCTDILISQKYNLLETAHVGEDHESEGITFLFKVKEGISKQS-FGIYCAKV 827
A. thaliana   MSH5    PVSEKIKFYTMSVLXPDTES-----------ANMEEIVFLYRLIPGQTLLS-YGLHCALL 741
C. elegans    MSH5    ETNIATFLTFTVLR--EAG---------------GKIKYLFRMTPGLVDCS-FALSVAKE 808
H. sapiens    MSH5    PQGPLVQYLTMETC--EDG---------------NDLVFFYQVCEGVAKASHASHTAAQA 762
M. musculus   MSH5    PQGPLVQYLTMETC--EDG---------------EDLVFFYQLCQGVASASHASHTAAQA 760
H. sapiens    MSH4    YPNVENMHFEVQHV--KNTS-----------RNKEAILYTYKLSKGLTEEKNYGLKAAEV 850
M. musculus   MSH4    YLNVENMHFEVQHV--KNTS-----------RNKDAILYTYKLSRGLTEEKNYGLKAAEA 872
A. thaliana   MSH4    YPNVKVLHFYVDIR--DNR-----------------LDFKFQLRDGTLHVPHYGLLLAEV 717
C. elegans    MSH4    SNAIDNYHFLPQTD--------------------ENSTKKHKLLRGQYRGPLYGFELVEL 751
S. cerevisiae MSH4    KPAVSHLHMDAVLLN------------------DNSVKMNYQLTQKSVAIENSGIRVVKK 798
```

```
                                ↓
S. cerevisiae MSH5    CGLSRDIVERA--EELSRMINRGDDVVQQCGNLTEKEMR-----------EFQKNQEIVK 874
A. thaliana   MSH5    AGVPEEVVKRA--AIVLDAFESNNNVDKLSLDKISS--------------QDQAFKDAVD 785
C. elegans    MSH5    EGIPPPVIGRA--CRIYKALKAGTLLKEIKAEVSNDNEKQLVEDMDVVLADEDGFMAAVE 866
H. sapiens    MSH5    G-LPDKLVARG--KEVSDLIRSGKPIKPVKDLLKKN--------------QMENCQTLVD 805
M. musculus   MSH5    G-LPDPLIARG--KEVSDLIRSGKPIKATNELLRRN--------------QMENCQALVD 803
H. sapiens    MSH4    SSLPPSIVLDA--KEITTQITRQILQNQRSTPEMER--------------QRAVYHLATR 894
M. musculus   MSH4    SSLPSSIVLDA--RDITTQITRQILQNQRSSPEMDR--------------QRAVYHLATR 916
A. thaliana   MSH4    AGLPSTVIDTA--RIITKRITDKENKRIELNCGKHH--------------EIHRIYRVAQ 761
C. elegans    MSH4    STIPDEVIEHA--QSLATELRANVEDTERDYDSERRRIK---------VYMNHRFRECAE 800
S. cerevisiae MSH4    IFNPDIIAEAYNIHSLLKIAKARTENEDSNGVVDQKTIN--------QMKRIHNLVAILK 850

                       ↓
S. cerevisiae MSH5    KFLSWDLDLETTTTSENLRLKLKNFLR--------------------------------- 901
A. thaliana   MSH5    KFAELDISK---GDIHAFFQDIFTS----------------------------------- 807
C. elegans    MSH5    SFVKRKKTSFCESSMRNVSEEIEKERSEASTPASKSRSTITARSNSVLSSRSMASVDQLS 926
H. sapiens    MSH5    KFMKLDLEDPNLD-LNVFMSQEVLPAATSIL----------------------------- 835
M. musculus   MSH5    KFLKLDLEDPTLD-LDIFISQEVLPAAPTIL----------------------------- 833
H. sapiens    MSH4    LVQTARNSQLDPDSLRIYLSNLKKKYKEDFPRTEQVPEKTEE------------------ 936
M. musculus   MSH4    LVQAARNSQLEPDRLRTYLSNLKKKYAGDFPRAVGLPEKTEE------------------ 958
A. thaliana   MSH4    RLICLKYSRQTEDSIRQALQNLNESFTEERL----------------------------- 792
C. elegans    MSH4    YFMDTHGEKWKEEKEAIDKMKALRKYLVDELAKIDSQEQMCQ------------------ 842
S. cerevisiae MSH4    ECAGNEKEPLTLGKLKEINSDFIENFEE-------------------------------- 878

S. cerevisiae MSH5    ------------------------------------------------------------
A. thaliana   MSH5    ------------------------------------------------------------
C. elegans    MSH5    VLDALLPKKKKKKVTGSSMESSMSPDPFQEEDEGTEGEEDQISAPVSRPTLPSVQKYASE 986
H. sapiens    MSH5    ------------------------------------------------------------
M. musculus   MSH5    ------------------------------------------------------------
H. sapiens    MSH4    ------------------------------------------------------------
M. musculus   MSH4    ------------------------------------------------------------
A. thaliana   MSH4    ------------------------------------------------------------
C. elegans    MSH4    ------------------------------------------------------------
S. cerevisiae MSH4    ------------------------------------------------------------

S. cerevisiae MSH5    ------------------------------------------------------------
A. thaliana   MSH5    ------------------------------------------------------------
C. elegans    MSH5    EEKQQSINSRHSFSTRTAIHIPTPIQMGEAGGVKRPRSTSTSSPGPSASKSVRTEVFKKT 1046
H. sapiens    MSH5    ------------------------------------------------------------
M. musculus   MSH5    ------------------------------------------------------------
H. sapiens    MSH4    ------------------------------------------------------------
M. musculus   MSH4    ------------------------------------------------------------
A. thaliana   MSH4    ------------------------------------------------------------
C. elegans    MSH4    ------------------------------------------------------------
S. cerevisiae MSH4    ------------------------------------------------------------

S. cerevisiae MSH5    ------------------------------------------------------------
A. thaliana   MSH5    ------------------------------------------------------------
C. elegans    MSH5    PNVKESQVLETPKQLSISSFLEPKFPSSEKDVISRVSERYLQSDPFKTPISDRRSQQSSR 1106
H. sapiens    MSH5    ------------------------------------------------------------
M. musculus   MSH5    ------------------------------------------------------------
H. sapiens    MSH4    ------------------------------------------------------------
M. musculus   MSH4    ------------------------------------------------------------
A. thaliana   MSH4    ------------------------------------------------------------
C. elegans    MSH4    ------------------------------------------------------------
S. cerevisiae MSH4    ------------------------------------------------------------
```

```
S. cerevisiae MSH5    ------------------------------------------------------------
A. thaliana   MSH5    ------------------------------------------------------------
C. elegans    MSH5    HSTPKNRSMNQSLIQSARDTPHETIRSSNEVNPEFFNIFNFPDDSILKSQDTYDPNVTPR 1166
H. sapiens    MSH5    ------------------------------------------------------------
M. musculus   MSH5    ------------------------------------------------------------
H. sapiens    MSH4    ------------------------------------------------------------
M. musculus   MSH4    ------------------------------------------------------------
A. thaliana   MSH4    ------------------------------------------------------------
C. elegans    MSH4    ------------------------------------------------------------
S. cerevisiae MSH4    ------------------------------------------------------------

S. cerevisiae MSH5    ------------------------------------------------------------
A. thaliana   MSH5    ------------------------------------------------------------
C. elegans    MSH5    SSSRRELRPDVSHSQNSQFGEVFSELGTQFSIFNSQQSFPGNSMGTTNPDCSIFDDFFAN 1226
H. sapiens    MSH5    ------------------------------------------------------------
M. musculus   MSH5    ------------------------------------------------------------
H. sapiens    MSH4    ------------------------------------------------------------
M. musculus   MSH4    ------------------------------------------------------------
A. thaliana   MSH4    ------------------------------------------------------------
C. elegans    MSH4    ------------------------------------------------------------
S. cerevisiae MSH4    ------------------------------------------------------------

S. cerevisiae MSH5    ------------------------------------------------------------
A. thaliana   MSH5    ------------------------------------------------------------
C. elegans    MSH5    SQDGEKKIDSTKTSMPIVNSDNFIFKTPEPRSSEKQRSLLKNKGQASNSSISPSSLILGQ 1286
H. sapiens    MSH5    ------------------------------------------------------------
M. musculus   MSH5    ------------------------------------------------------------
H. sapiens    MSH4    ------------------------------------------------------------
M. musculus   MSH4    ------------------------------------------------------------
A. thaliana   MSH4    ------------------------------------------------------------
C. elegans    MSH4    ------------------------------------------------------------
S. cerevisiae MSH4    ------------------------------------------------------------

S. cerevisiae MSH5    ------------------------------------------------------------
A. thaliana   MSH5    ------------------------------------------------------------
C. elegans    MSH5    LAFGDVDQTPRPRGDNPIEFQYDVVDDDDPIFEEKNCSAPVFEFLKSNDDEEDDEFLKSF 1346
H. sapiens    MSH5    ------------------------------------------------------------
M. musculus   MSH5    ------------------------------------------------------------
H. sapiens    MSH4    ------------------------------------------------------------
M. musculus   MSH4    ------------------------------------------------------------
A. thaliana   MSH4    ------------------------------------------------------------
C. elegans    MSH4    ------------------------------------------------------------
S. cerevisiae MSH4    ------------------------------------------------------------

S. cerevisiae MSH5    -----------------------
A. thaliana   MSH5    -----------------------
C. elegans    MSH5    LETEGSLHIDTSADETIDRSKRS 1369
H. sapiens    MSH5    -----------------------
M. musculus   MSH5    -----------------------
H. sapiens    MSH4    -----------------------
M. musculus   MSH4    -----------------------
A. thaliana   MSH4    -----------------------
C. elegans    MSH4    -----------------------
S. cerevisiae MSH4    -----------------------
```
