# Supplementary figures and images for: Genetic Analysis of Baker's Yeast Msh4-Msh5 Reveals a Threshold Crossover Level for Meiotic Viability
Source: PLoS Genet. 2010 Aug 26;6(8):e1001083. doi: 10.1371/journal.pgen.1001083 (PMC2928781; doi:10.1371/journal.pgen.1001083)

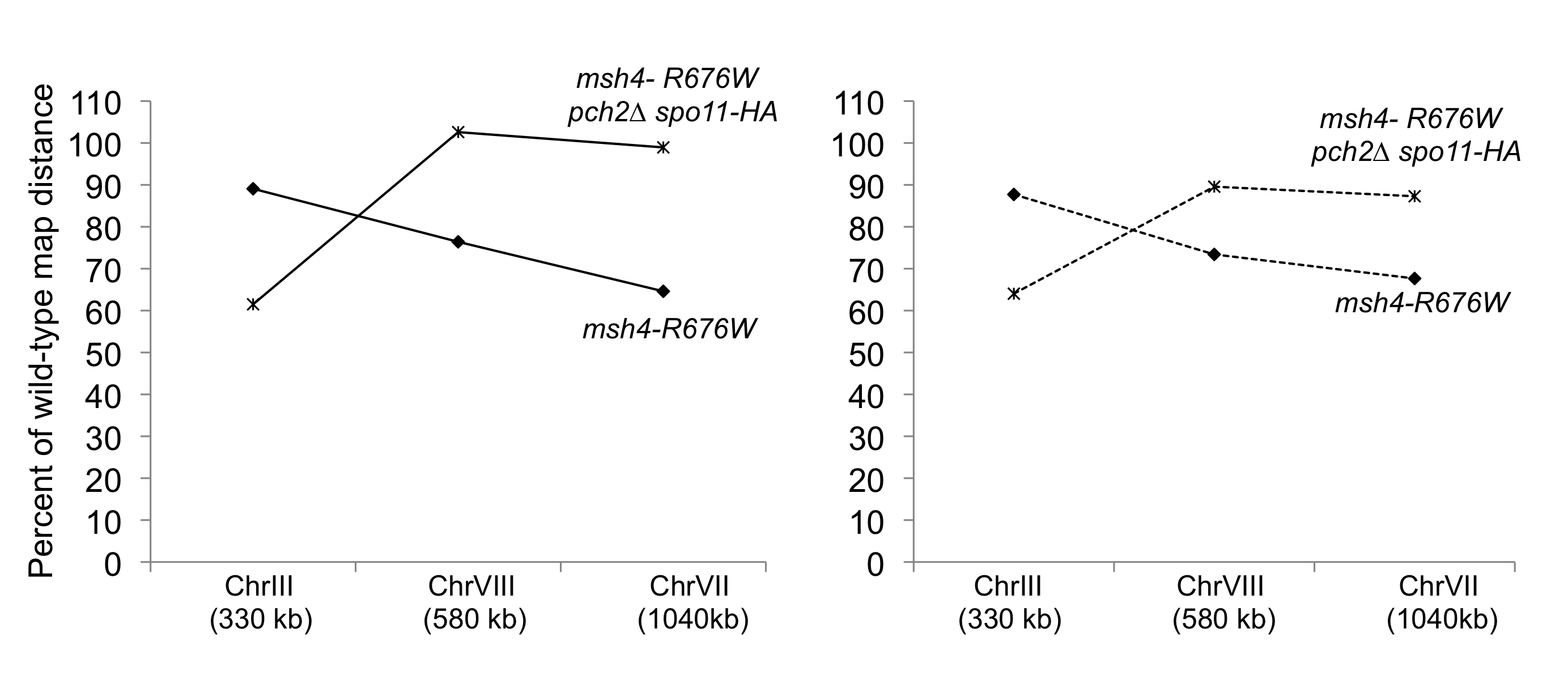

Supplement: Figure S2 — Comparison of the crossover distribution on chromosomes III, VII and VIII in msh4/5-R676W versus the msh4-R676W pch2Δ spo11-HA triple mutant. Distribution of crossovers from tetrads (left panel) and spores (right panel) across chromosomes III, VII and VIII in the NHY strain background is shown for the msh4-R676W and the msh4-R676W pch2Δ spo11-HA triple mutant as a percent of wild-type map distance. (0.23 MB TIF) [file pgen.1001083.s002.tif]

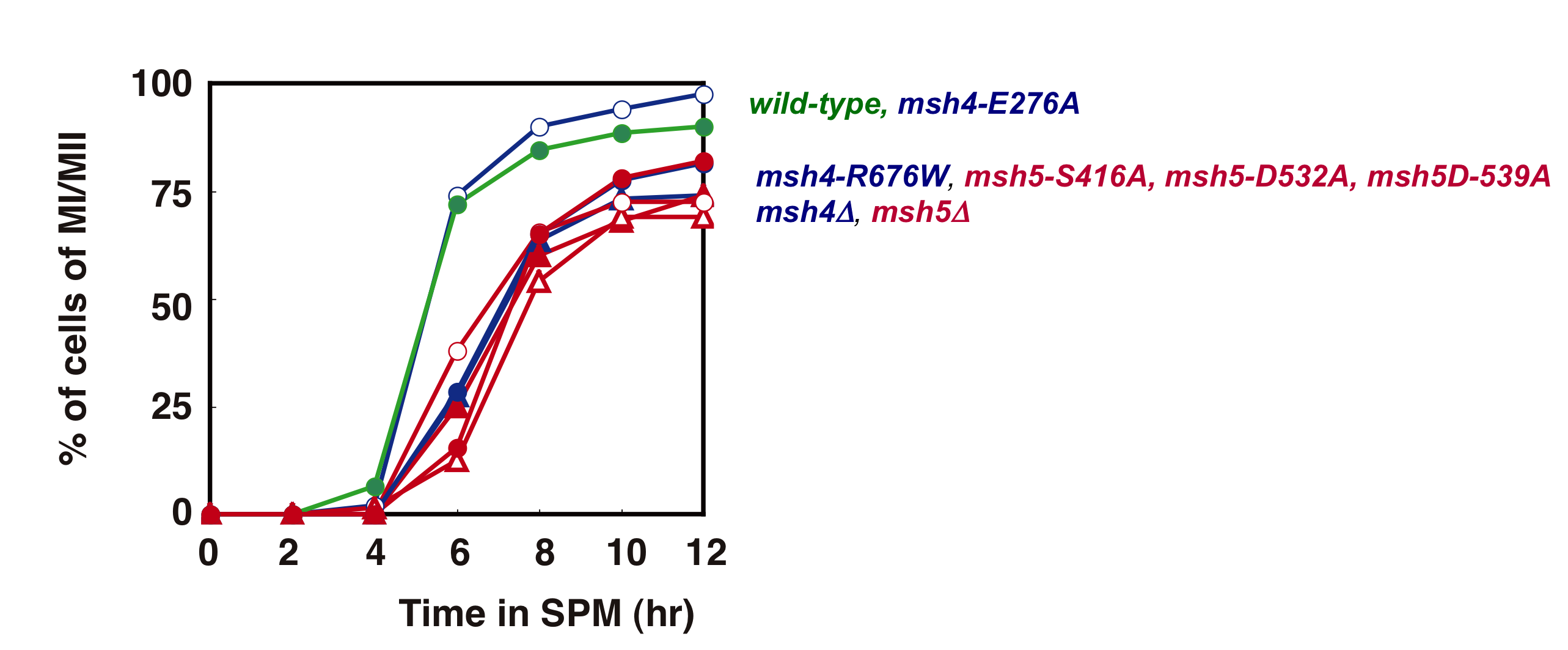

Supplement: Figure S3 — Analysis of meiotic divisions in msh4/5-t and msh4/5-bt cells. Synchronized meiotic cultures of wild-type and msh4Δ, msh5Δ, msh4/5-t (msh4-E276A, msh4-R676W, msh5-S416A, msh5-D539A) and msh4/5-bt (msh5-D532A) mutants (strains examined in Figure 7) were analyzed for the completion of at least MI (MI/MII) as measured by DAPI staining. A representative experiment is shown. (0.26 MB TIF) [file pgen.1001083.s003.tif]
