# Supplementary material for: Genetic Analysis of Baker's Yeast Msh4-Msh5 Reveals a Threshold Crossover Level for Meiotic Viability
Source: PLoS Genet. 2010 Aug 26;6(8):e1001083. doi: 10.1371/journal.pgen.1001083 (PMC2928781; doi:10.1371/journal.pgen.1001083)
Supplement: Table S1 — Strains used in this study. The Plasmid column refers to MSH4/5::KANMX and msh4/5::KANMX integration vectors used to make the indicated EAY1108 derivative strains. (0.10 MB DOC) [file pgen.1001083.s004.doc]

**Table S1. Strains used in this study**

| **Strain name** | **Genotype** | **Plasmid** | **Source** |
| --- | --- | --- | --- |
|  | **SK1 congenic** |  |  |
| EAY1108 | *MATa*, *ho::hisG*, *lys2*, *ura3*, *leu2::hisG*, *trp1::hisG*,  *URA3*-cenXVi, *LEU2*-chXVi, *LYS2*-chXVi |  | [24] |
| EAY2409 | as EAY1108 except *msh4Δ::NATMX4* |  | This work |
| EAY1281 | as EAY1108 except *msh5Δ::NATMX4* |  | [24] |
| EAY2417 | as EAY1108 except *MSH5::KANMX4* | pEAA424 | This work |
| EAY2419 | as EAY1108 except *MSH4::KANMX4* | pEAA427 | This work |
| EAY2421 | as EAY1108 except *msh5-E45A::KANMX4* | pEAA460 | This work |
| EAY2423 | as EAY1108 except *msh5-D76A::KANMX4* | pEAA461 | This work |
| EAY2425 | as EAY1108 except *msh5-E135A::KANMX4* | pEAA462 | This work |
| EAY2427 | as EAY1108 except *msh5-D147A::KANMX4* | pEAA463 | This work |
| EAY2429 | as EAY1108 except *msh5-F161A::KANMX4* | pEAA464 | This work |
| EAY2431 | as EAY1108 except *msh5-N182A::KANMX4* | pEAA465 | This work |
| EAY2433 | as EAY1108 except *msh5-D250A::KANMX4* | pEAA466 | This work |
| EAY2435 | as EAY1108 except *msh5-W298A::KANMX4* | pEAA467 | This work |
| EAY2437 | as EAY1108 except *msh5-S416A::KANMX4* | pEAA468 | This work |
| EAY2439 | as EAY1108 except *msh5-T423A::KANMX4* | pEAA469 | This work |
| EAY2441 | as EAY1108 except *msh5-D433A::KANMX4* | pEAA470 | This work |
| EAY2443 | as EAY1108 except *msh5-R436A::KANMX4* | pEAA471 | This work |
| EAY2445 | as EAY1108 except *msh5-Y480A::KANMX4* | pEAA472 | This work |
| EAY2447 | as EAY1108 except *msh5-Y486A::KANMX4* | pEAA473 | This work |
| EAY2449 | as EAY1108 except *msh5-E495A::KANMX4* | pEAA474 | This work |
| EAY2451 | as EAY1108 except *msh5-D527A::KANMX4* | pEAA475 | This work |
| EAY2453 | as EAY1108 except *msh5-D532A::KANMX4* | pEAA476 | This work |
| EAY2455 | as EAY1108 except *msh5-D539A::KANMX4* | pEAA477 | This work |
| EAY2457 | as EAY1108 except *msh5-Y661A::KANMX4* | pEAA478 | This work |
| EAY2459 | as EAY1108 except *msh5-D680A::KANMX4* | pEAA479 | This work |
| EAY2461 | as EAY1108 except *msh5-R837A::KANMX4* | pEAA480 | This work |
| EAY2463 | as EAY1108 except *msh5-F876A::KANMX4* | pEAA481 | This work |
| EAY2465 | as EAY1108 except *msh5-V488A::KANMX4* | pEAA492 | This work |
| EAY2467 | as EAY1108 except *msh5-I490A::KANMX4* | pEAA493 | This work |
| EAY2469 | as EAY1108 except *msh5-I537A::KANMX4* | pEAA494 | This work |
| EAY2471 | as EAY1108 except *msh5-L548A::KANMX4* | pEAA495 | This work |
| EAY2473 | as EAY1108 except *msh5-G648A::KANMX4* | pEAA496 | This work |
| EAY2475 | as EAY1108 except *msh5-R685W::KANMX4* | pEAA497 | This work |
| EAY2477 | as EAY1108 except *msh4-E111A::KANMX4* | pEAA438 | This work |
| EAY2479 | as EAY1108 except *msh4-N126A::KANMX4* | pEAA439 | This work |
| EAY2480 | as EAY1108 except *msh4-D139A::KANMX4* | pEAA440 | This work |
| EAY2482 | as EAY1108 except *msh4-Y143A::KANMX4* | pEAA441 | This work |
| EAY2898 | as EAY1108 except *msh4-F194A::KANMX4* | pEAA442 | This work |
| EAY2484 | as EAY1108 except *msh4-N195A::KANMX4* | pEAA443 | This work |
| EAY2486 | as EAY1108 except *msh4-D210A::KANMX4* | pEAA444 | This work |
| EAY2487 | as EAY1108 except *msh4-D268A::KANMX4* | pEAA445 | This work |
| EAY2489 | as EAY1108 except *msh4-E276A::KANMX4* | pEAA446 | This work |
| EAY2491 | as EAY1108 except *msh4-E324A::KANMX4* | pEAA447 | This work |
| EAY2493 | as EAY1108 except *msh4-E328A::KANMX4* | pEAA448 | This work |
| EAY2495 | as EAY1108 except *msh4-N409A::KANMX4* | pEAA449 | This work |
| EAY2900 | as EAY1108 except *msh4-E425A::KANMX4* | pEAA450 | This work |
| EAY2499 | as EAY1108 except *msh4-D453A::KANMX4* | pEAA451 | This work |
| EAY2501 | as EAY1108 except *msh4-R456A::KANMX4* | pEAA452 | This work |
| EAY2503 | as EAY1108 except *msh4-E461A::KANMX4* | pEAA453 | This work |
| EAY2505 | as EAY1108 except *msh4-F491A::KANMX4* | pEAA454 | This work |
| EAY2507 | as EAY1108 except *msh4-N532A::KANMX4* | pEAA455 | This work |
| EAY2509 | as EAY1108 except *msh4-R534A::KANMX4* | pEAA456 | This work |
| EAY2511 | as EAY1108 except *msh4-E732A::KANMX4* | pEAA457 | This work |
| EAY2513 | as EAY1108 except *msh4-H764A::KANMX4* | pEAA458 | This work |
| EAY2515 | as EAY1108 except *msh4-D772A::KANMX4* | pEAA459 | This work |
| EAY2517 | as EAY1108 except *msh4-Y485A::KANMX4* | pEAA484 | This work |
| EAY2519 | as EAY1108 except *msh4-L493A::KANMX4* | pEAA485 | This work |
| EAY2521 | as EAY1108 except *msh4-I495A::KANMX4* | pEAA486 | This work |
| EAY2523 | as EAY1108 except *msh4-I542A::KANMX4* | pEAA487 | This work |
| EAY2525 | as EAY1108 except *msh4-L553A::KANMX4* | pEAA488 | This work |
| EAY2527 | as EAY1108 except *msh4-G639A::KANMX4* | pEAA489 | This work |
| EAY2529 | as EAY1108 except *msh4-R676W::KANMX4* | pEAA490 | This work |
|  |  |  |  |
| EAY1112 | *MATα*, *ho::hisG*, *lys2*, *ura3*, *leu2::hisG*, *trp1::hisG*,  *ade2::hisG*, *his3::hisG*, *TRP1*-cenXVi |  | [24] |
| EAY2411 | as EAY1112 except *msh4Δ::NATMX4* |  | This work |
| EAY1280 | as EAY1112 except *msh5Δ::NATMX4* |  | [24] |
|  | **SK1 isogenic** |  |  |
| NHY942 | *MATα, ho::hisG, ade2∆, can1, ura3(∆Sma-Pst),*  *met13-B, trp5-S, CEN8::URA3, thr1-A, cup1^s^* |  | [23] |
| EAY2843 | as NHY942 except *msh4Δ::NATMX4* |  | This work |
| EAY2846 | as NHY942 except *msh5Δ::NATMX4* |  | This work |
| EAY2705 | as NHY942 except *msh4Δ::NATMX4*,  *spo11-HA3HIS6::KANMX4* |  | This work |
| EAY2719 | as NHY942 except *msh4Δ::NATMX4*, *pch2Δ::NATMX4* |  | This work |
| EAY2777 | as NHY942 except *msh4Δ::NATMX4*, *pch2Δ::NATMX4*, |  | This work |
|  | *spo11-HA3HIS6::KANMX4* |  |  |
| NHY943 | *MATa, ho::hisG, ade2∆, ura3(∆Sma-Pst), leu2::hisG,*  *CEN3::ADE2, lys5-P, cyh2^r^, his4-B* |  | [23] |
| EAY2844 | as NHY943 except *msh4Δ::NATMX4* |  | This work |
| EAY2848 | as NHY943 except *msh5Δ::NATMX4* |  | This work |
| EAY2849 | as NHY943 except *msh4-E276A::KANMX4* |  | This work |
| EAY2851 | as NHY943 except *msh4-R676W::KANMX4* |  | This work |
| EAY2855 | as NHY943 except *msh5-S416A::KANMX4* |  | This work |
| EAY2857 | as NHY943 except *msh5-D539A::KANMX4* |  | This work |
| EAY2688 | as NHY943 except *msh4-E276A::KANMX4,*  *pch2Δ::NATMX4* |  | This work |
| EAY2700 | as NHY943 except *msh4-E276A::KANMX4,*  *spo11-HA3HIS6::KANMX4* |  | This work |
| EAY2780 | as NHY943 except *msh4-E276A::KANMX4,*  *pch2Δ::NATMX4,* *spo11- HA3HIS6::KANMX4* |  | This work |
| EAY2785 | as NHY943 except *msh5-D532A::KANMX4* |  | This work |
| EAY2691 | as NHY943 except *msh4-R676W::KANMX4,*  *pch2Δ::NATMX4* |  | This work |
| EAY2703 | as NHY943 except *msh4-R676W::KANMX4,*  *spo11-HA3HIS6::KANMX4* |  | This work |
| EAY2782 | as NHY943 except *msh4-R676W::KANMX4,*  *pch2Δ::NATMX4*, *spo11-HA3HIS6::KANMX4* |  | This work |
